# Geographic Variations in Cardiometabolic Risk Factors in Luxembourg

**DOI:** 10.3390/ijerph14060648

**Published:** 2017-06-16

**Authors:** Ala’a Alkerwi, Illiasse El Bahi, Saverio Stranges, Jean Beissel, Charles Delagardelle, Stephanie Noppe, Ngianga-Bakwin Kandala

**Affiliations:** 1 Luxembourg Institute of Health (LIH), Department of Population Health, Epidemiology and Public Health Research Unit EPHRU, Strassen, L-1445 Strassen Luxembourg City, Luxembourg; Alaa.AlKerwi@lih.lu (A.A.); Illiasse.ElBahi@lih.lu (I.E.B.); saverio.stranges@uwo.ca (S.S.); 2 Department of Epidemiology & Biostatistics, Schulich School of Medicine & Dentistry, Western University, London, ON N6A 5C1, Canada; 3 Centre Hospitalier du Luxembourg, Grand-Duchy of Luxembourg, 1210 Luxembourg City, Luxembourg; beissel.jean@chl.lu (J.B.); Delagardelle.Charles@chl.lu (C.D.); Noppe.Stephanie@chl.lu (S.N.); 4 Department of Mathematics, Physics and Electrical Engineering, Faculty of Engineering and Environment, Northumbria University, Newcastle upon Tyne NE1 8ST, UK; 5 Faculty of Health and Sport Sciences, University of Agder, Postboks 422, 4604 Kristiansand, Norway

**Keywords:** cardio metabolic, hypertension, geographic variation, Luxembourg

## Abstract

Cardiovascular disease (CVD) and associated behavioural and metabolic risk factors constitute a major public health concern at a global level. Many reports worldwide have documented different risk profiles for populations with demographic variations. The objective of this study was to examine geographic variations in the top leading cardio metabolic and behavioural risk factors in Luxembourg, in order to provide an overall picture of CVD burden across the country. The analysis conducted was based on data from the nationwide ORISCAV-LUX survey, including 1432 subjects, aged 18–69 years. A self-reported questionnaire, physical examination and blood sampling were performed. Age and sex-adjusted risk profile maps were generated using multivariate Bayesian geo-additive regression models, based on Markov Chain Monte Carlo techniques and were used to evaluate the significance of the spatial effects on the distribution of a range of cardio metabolic risk factors, namely smoking, high body mass index (BMI), high blood pressure, high fasting plasma glucose, alcohol use, high total cholesterol, low glomerular filtration rate, and physical inactivity. Higher prevalence of smoking was observed in the northern regions, higher overweight/obesity and abdominal obesity clustered in the central belt, whereas hypertension was spotted particularly in the southern part of the country. Maps revealed that subjects residing in Luxembourg canton were significantly less likely to be hypertensive or overweight/obese, whereas they were less likely to practice physical activity of ≥8000 Metabolic Equivalent of Task (MET)-min/week. These patterns were also observed at the municipality level in Luxembourg. Statistically, there were non-significant spatial patterns regarding smoking, diabetes, total serum cholesterol and low glomerular filtration rate risk distribution. This comprehensive risk profile mapping showed remarkable geographic variations in cardio metabolic and behavioural risk factors. Considering the prominent burden of CVD this research provides opportunities for tailored interventions and may help to better fight against this escalating public health problem.

## 1. Introduction

Cardiovascular disease (CVD) and associated behavioural and metabolic risk factors constitute a major public health concern not only in Europe and Luxembourg but at a global level. In 2010, ischemic heart disease and stroke collectively killed 12.9 million people or one in four deaths worldwide, compared with one in five in 1990; 1.3 million deaths were due to diabetes, which doubled since 1990 [1]. These diseases, which primarily cause avoidable premature death, were among the top 15 causes of Disability-Adjusted Life-Years (DALYs) globally [2].

Additionally, the Global Burden of Disease, Injuries, and Risk Factor study in 2013 (GBD 2013) identified that smoking, high body mass index (BMI), high blood pressure (BP), high fasting plasma glucose (FPG), alcohol use, high total cholesterol (TC), low glomerular filtration rate (GFR), physical inactivity, drug use and high intake of processed meat were the top ten leading causes of DALYs for both sexes in Luxembourg [3]. This national pattern converged with most high-income countries, calling attention to the important impact of these diseases and associated pathologies on population health, individual and societal well-being, as well as to their burden on national health care system.

Previous research has demonstrated substantial variations in CVD burden. Many country-specific reports have documented different risk profiles for populations with demographic variations [4,5,6,7,8]. In Luxembourg, the most recent national estimates for the prevalence of hypertension, diabetes, dyslipidaemia, smoking and obesity for adults were at 34.5%, 4.4%, 69.9%, 22.3% and 20.9%, respectively [8]. All prevalence rates increased with age (except smoking), with marked gender differences (except diabetes). Furthermore, there was a significant difference in the prevalence of hypertension and lipid disorders by subject’s country of birth [8].

Recently, evidence regarding geographic variations in cardiovascular risk profiles was reported among provinces in Canada [9], among women in cities of the United States [10], between southern and northern populations of China [11], in Malaysia [12] and in African populations [13,14]. Studies have found that risk factors tend to cluster within socially disadvantaged regions. After controlling for socioeconomic status (SES), regional effects do exist, although tend to be small compared to individual effects related to lifestyle [9,15]. Research also indicates that no single parameter can explain differences driven by the geographic location of a community. Jarvie et al*.* underlined that multiple factors make up a community, such as racial and ethnic majorities, education, and income, in addition to local conditions that affect lifestyle, such as climate, grocery stores, transportation, safety and availability of parks and open spaces. Therefore, regional variation is likely to reflect the complex interplay between a region and the inhabiting population [10].

While the distribution of cardiovascular risk factors [8] and risk profile variations [16] have been well documented in Luxembourg, information regarding the geographical variation has not yet been reported. Most public health research has focused on person and time, with little consideration of the implications of space dimension on disease processes [17,18,19]. However, evidence regarding the concentration of a health problem in identifiable places or searching for geographical disease clustering is essential for an efficient distribution of resources for prevention and treatment [3]. Therefore, from the perspective of public health practice, understanding the overall picture of CVD burden in Luxembourg provides opportunities for tailored interventions to improve CVD outcomes. The aim of this study was to examine geographic variations in the top leading behavioural and cardio metabolic risk factors [3] among the adult population of residents in Luxembourg by using data from the Observation of Cardiovascular Risk Factors in Luxembourg (ORISCAV-LUX) survey.

## 2. Methods

### 2.1. Studied Population

The ORISCAV-LUX survey, conducted in 2007–2008, is the first population-based study to monitor cardiovascular risk factors among adults, aged 18 to 69 years, in Luxembourg. This survey involved a self-reported questionnaire that covered socio-demographic aspects, lifestyle and personal health problems. In addition, blood pressure, body weight, height, waist circumference, as well as blood and urine sampling were performed by well-trained nurses. Further details regarding the measurement of anthropometric and biochemical parameters are available elsewhere [8,20,21].

The ORISCAV-LUX study employed a stratified random sampling design proportionate to the population size, according to age, sex and geographical districts (Luxembourg, Diekirch and Grevenmacher). A detailed description of sampling procedures and sample representativeness was published elsewhere [22]. Briefly, 1432 participants were recruited, from which two non-residents in Luxembourg were excluded from the present analyses.

The ORISCAV-LUX study was conducted according to the guidelines laid down in the Declaration of Helsinki. All procedures involving human subjects were approved by the National Research Ethics Committee (N200609/03) and the National Commission for Private Data Protection. Written informed consent was obtained from all subjects (copy available on need). This document has been written in line with our national legal authorities’ guidelines.

### 2.2. Outcome Measurement

Based on findings from the GBD study and considering data availability, we focused on the following top leading behavioural and metabolic risk factors, each identified as binary outcomes: smoking (current/former smoker vs. never smoked), high BMI (overweight/obesity with BMI > 25 vs. ≤25), high BP (BP of ≥140 mmHg and/or DBP ≥ 90 mmHg and/or antihypertensive medications intake vs. no hypertension), high FPG (FPG of ≥126 mg/dL and/or antidiabetic medications intake vs. no diabetes), alcohol use (daily alcohol drinkers vs. non-drinkers), high TC (total cholesterol >4.8 mmol/L vs. equal or below this level), low GFR (eGFR <60 mL per min per 1.73 m^2^ vs. equal or above this level) and physical inactivity (when physical activity was <8000 Metabolic Equivalent of Task (MET) min per week vs. equal or above this level). Owing to the high impact of abdominal obesity on cardio metabolic health, we also considered this factor in our analyses, defined as binary variable (waist circumference (WC) ≥ 102 cm for men and ≥ 88 cm for women vs. lower than these levels). Table 1.

### 2.3. Geographical Variables

**Figure 1:**
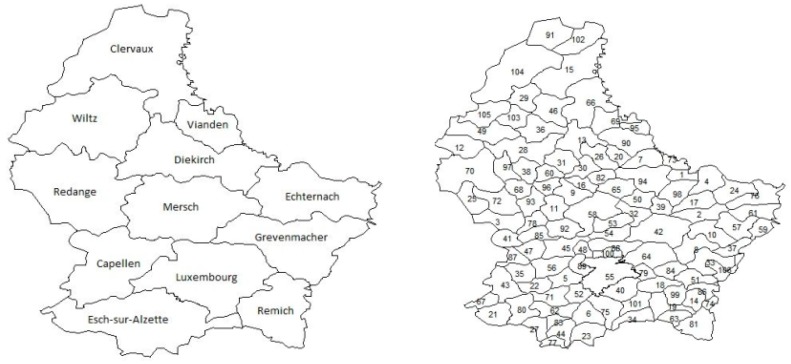
Map of Luxembourg by cantons (left) and municipalities (right). 1, Beaufort. 2, Bech. 3, Beckerich. 4, Berdorf. 5, Bertrange. 6, Bettembourg. 7, Bettendorf. 8, Betzdorf. 9, Bissen. 10, Biwer. 11, Boevange-sur-Attert. 12, Boulaide. 13, Bourscheid. 14, Bous. 15, Clervaux. 16, Colmar-Berg. 17, Consdorf. 18, Contern. 19, Dalheim. 20, Diekirch. 21, Differdange. 22, Dippach. 23, Dudelange. 24, Echternach. 25, Ell. 26, Erpeldange. 27, Esch-sur-Alzette. 28, Esch-sur-Sure. 29, Eschweiler. 30, Ettelbruck. 31, Feulen. 32, Fischbach. 33, Flaxweiler. 34, Frisange. 35, Garnich. 36, Goesdorf. 37, Grevenmacher. 38, Grosbous. 39, Heffingen. 40, Hesperange. 41, Hobscheid. 42, Junglinster. 43, Kaerjen. 44, Kayl. 45, Kehlen. 46, Kiischpelt. 47, Koerich. 48, Kopstal. 49, Lac De La Haute Sure. 50, Larochette. 51, Lenningen. 52, Leudelange. 53, Lintgen. 54, Lorentzweiler. 55, Luxembourg. 56, Mamer. 57, Manternach. 58, Mersch. 59, Mertert. 60, Mertzig. 61, Mompach. 62, Mondercange. 63, Mondorf-Les-Bains. 64, Niederanven. 65, Nommern. 66, Parc Hosingen. 67, Petange. 68, Preizerdaul. 69, Putscheid. 70, Rambrouch. 71, Reckange-sur-Mess. 72, Redange. 73, Reisdorf. 74, Remich. 75, Roeser. 76, Rosport. 77, Rumelange. 78, Saeul. 79, Sandweiler. 80, Sanem. 81, Schengen. 82, Schieren. 83, Schifflange. 84, Schuttrange. 85, Septfontaines. 86, Stadtbredimus. 87, Steinfort. 88, Steinsel. 89, Strassen. 90, Tandel. 91, Troisvierges. 92, Tuntange. 93, Useldange. 94, Vallée de L'Ernz. 95, Vianden. 96, Vichten. 97, Wahl. 98, Waldbillig. 99, Waldbredimus. 100, Walferdange. 101, Weiler-la-Tour. 102, Weiswampach. 103, Wiltz. 104, Wincrange. 105, Winseler. 106, Wormeldange.

The independent “explanatory” variable was the participant’s geographic location, measured in terms of the canton or municipality of residence at the time of the survey. Note that Luxembourg is administratively divided into 12 cantons and 106 municipalities (Figure 1).

### 2.4. Statistical Analysis

First, a descriptive analysis of behavioural and metabolic risk factors of the participants was performed according to sex and then according to canton and municipality. The statistical significance of probable associations between the geographic location (canton and municipality) and each behavioural and cardio metabolic risk factor was assessed using chi-square (χ^2^) and Mann-Whitney U-tests, as appropriate.

Then, to account for geographical effects on the prevalence of each risk factor, we employed a fully Bayesian approach using Markov Chain Monte Carlo (MCMC) techniques for inference and model checking [28]. This was achieved using multivariate Bayesian geo-additive regression models adjusted for age and gender; the effect of age on each risk factor was assumed to be linear. The standard measure of effect was the posterior odds ratio (POR) and its 95% confidence interval (CI). Age- and gender-adjusted odds ratios (ORs) of each risk factor were computed using logistic regression models, with women and Luxembourg (at the canton and municipality level) as reference categories. Age- and sex-adjusted total residual spatial effects were generated and presented on smoothed maps using graduated colouring to represent the risk for each risk factor. To account for the stratified random sampling method used to recruit the participants, weighted statistical methods were applied to produce nationally representative estimates.

All descriptive statistical analyses were performed using PASW^®^ for Windows^®^ version 21.0 software (formerly SPSS Statistics Inc., New York, NY, USA). BayesX software package^®^ (University of Munich, Munich, Germany) version 3.0.2, which permits Bayesian inference based on MCMC simulation techniques which was used to map the geographic distribution of the top leading cardio metabolic risk factors of interest.

## 3. Results

### 3.1. Subjects’ Characteristics

The ORISCAV-LUX sample consisted of 1430 participants including 734 (49.6%) women. Overall mean age of participants was 42.02 ± 0.04. There were significant sex-specific differences in the prevalence estimates of tobacco consumption (*p*-value = 0.04), alcohol use, hypertension and adiposity, where the prevalence estimates were higher in men, except for abdominal obesity (all *p*-value < 0.001). Age- and sex-adjusted prevalence estimates of smoking, high BMI (overweight/obesity), high abdominal obesity, hypertension, diabetes, alcohol use, high TC, low eGFR and low physical activity were 22.3%, 53.9%, 29.9%, 34.6%, 4.4%, 82.5%, 60.2%, 1.5% and 89.5%, respectively (Table 2).

### 3.2. Geographical Distribution of Top Leading Behavioural and Metabolic Risk Factors

**Figure 2:**
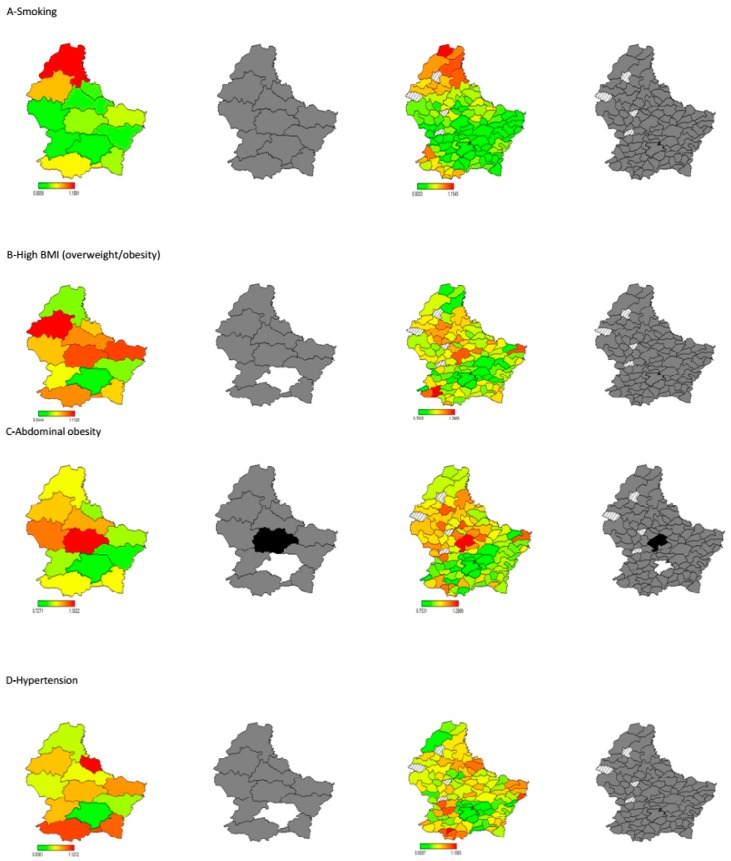

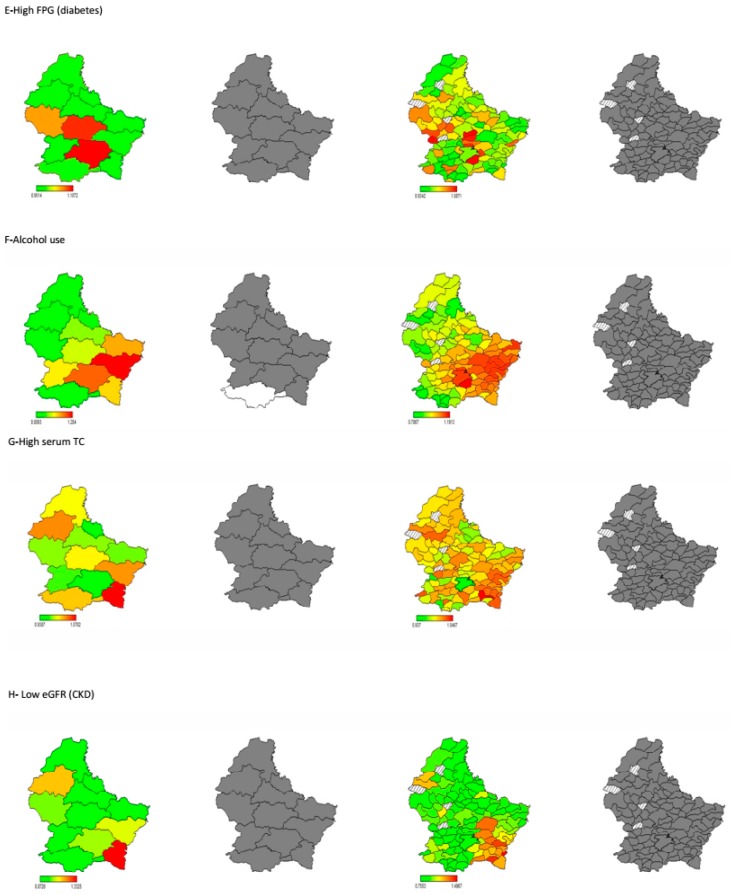

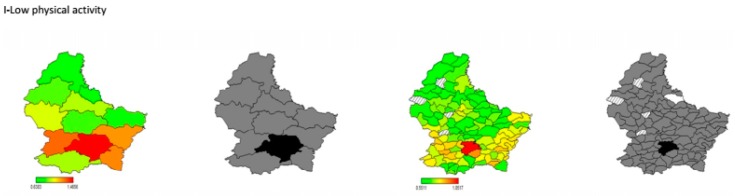
Gender- and age-adjusted total residual spatial effects of the metabolic risk factors at the canton and commune level in Luxembourg. Shown are the posterior odds ratios (red colour for a high risk and green colour for low risk) with their corresponding posterior probabilities at 80% nominal level (black colour for positive significant spatial effect, white colour for negative significant spatial effect, grey colour for non-significant spatial effect) and straight-lines segments indicating no data collected from these municipalities (four municipalities).

Figure 2A–I depicts in maps the age- and sex-adjusted total spatial effect of each risk factor of interest at the canton and commune level with the corresponding POR. The change of coloration shows the pattern of the spatial risk across the regions, with red colour indicating the highest risk (highest values of POR) whereas the green colour indicates the lowest risk (lowest values of POR). Next to each map representing the PORs are the corresponding posterior probabilities at 80% nominal level, with black colour indicating a positive significant spatial effect, white indicating a negative significant spatial effect, grey indicating a non-significant effect, and straight-lines segments indicating no data collected from these municipalities (four municipalities). (Supplementary Tables S1–S18 show reference tables estimating the crude prevalence of each risk factor and respective age- and sex-adjusted OR and POR).

#### 3.2.1. Smoking

Although statistically not significant, smoking prevalence estimates were different across cantons, ranging from 10.1% in Vianden to 38.1% in Clervaux and also across municipalities.

Taking account of the spatial effect, the highest risk was noted in the northern canton of the country, specifically in Clervaux (Posterior Odds Ratio (POR)) and 95% Confidence Interval (CI): 1.19 (0.88, 1.90) and in Troisvierges (1.15 (0.78, 2.16)) at the municipality level. Subjects residing in Luxembourg canton (0.89 (0.68, 1.09)) and municipality (0.92 (0.69, 1.13)) were at the lowest smoking risk (Figure 2A; Tables S1 and S2).

#### 3.2.2. High BMI

A notable prevalence of overweight/obesity was observed in most of the cantons, ranging from 44.9% in Clerveaux to 65.1% in Echternach (*p* = 0.16), as well as across municipalities (*p* = 0.05).

Considering the spatial effect, the highest likelihood of having high BMI was noted in the canton of Wiltz (1.11 (0.84, 1.61)) and in the municipality of Sanem (1.27 (0.89, 2.22)). A significant negative spatial effect (associated with a reduced risk of being overweight or obese) was observed in Luxembourg canton (0.84 (0.64, 1.03)) (Figure 2B; Tables S3 and S4).

#### 3.2.3. Abdominal Obesity

A significant variation in the prevalence estimate of abdominal obesity was observed across cantons (*p* = 0.02), ranging from 23.0% in Luxembourg to 47.6% in Redange.

Considering the spatial effect, the highest risk was noted in the canton and municipality of Mersch (1.30 (0.93, 2.06)) and (1.29 (0.89, 2.20)), respectively. However, a significant negative spatial effect (associated with a decreased risk of abdominal obesity) was observed in the canton and municipality of Luxembourg (0.73 (0.53, 0.96)) and (0.75 (0.53, 0.99)), respectively (Figure 2C; Tables S5 and S6).

#### 3.2.4. Hypertension

The crude prevalence of hypertension ranged from the lowest in Clervaux (27.6%) to the highest in Vianden (66.0%). This difference was only significant across municipalities (*p* = 0.04).

The maps presenting the spatial effect for hypertension showed the highest POR in the cantons of Vianden (1.12 (0.78, 1.04)), followed by Esch-sur-Alzette (1.08 (0.87, 1.37)) and Remich (1.07 (0.79, 1.57)). Subjects living in the Southern Schifflange municipality (1.16 (0.80, 1.88)) were also more likely to be hypertensive. However, a significant negative spatial effect was observed (lowest risk) in the canton and municipality of Luxembourg (0.84 (0.62, 1.04)), respectively (Figure 2D; Tables S7 and S8).

#### 3.2.5. Diabetes

There were no significant differences in the crude prevalence of diabetes across cantons (*p* = 0.77) and municipalities (*p* = 0.91). Taking into consideration the spatial effect, no remarkable variations of diabetes risk across cantons and municipalities were observed (Figure 2E; Tables S9 and S10).

#### 3.2.6. Alcohol Use

A high prevalence of alcohol use was registered across cantons (ranging from 90.6% in Grevenmacher to 71.0% in Redange) and municipalities, although differences were not statistically significant (*p* > 0.05).

Maps for spatial effects showed that subjects living in the southeastern region of the country, specifically in Grevenmacher canton (1.28 (0.90, 2.31)), were more likely to consume alcohol, whereas a significant negative spatial effect (reduced risk of alcohol use) was observed in Esch-sur-Alzette (0.79 (0.49, 1.12)) (Figure 2F; Tables S11 and S12).

#### 3.2.7. High Serum Total Cholesterol

Though not statistically significant, a global raised prevalence estimate of hypercholesterolemia were observed across cantons and municipalities, ranging from the lowest in Vianden (49.3%) to the highest in Remich (71.7%). Maps-G for spatial effect also showed non-significant variation across cantons and municipalities, although the highest POR was noted in Remich (1.08 (0.86, 1.50)) at the canton level and in Dalheim (1.05 (0.84, 1.39)) at the municipality level (Figure 2G; Tables S13 and S14).

#### 3.2.8. Low eGFR

Overall, there were 24 participants with eGFR lower than 60 mL per min per 1.73 m^2^, constituting a crude prevalence estimate of 1.5%. Maps-H spotted the highest POR in the canton of Remich (1.37 (0.67, 6.08)) and the lowest in Diekirch (0.83 (0.22, 1.57)) (Figure 2H; Tables S15 and S16).

#### 3.2.9. Low Physical Activity

There was significant variation in the practice of physical activity (equal or more than 8000 MET-min/week) across cantons and municipalities (*p* = 0.03 and *p* < 0.001, respectively), with the lowest prevalence estimates in the Southern central belt (Luxembourg, Remich, Cappellen) compared to the Northern regions of the country. Considering the spatial effect, map-I displayed a significant positive spatial effect (increased risk of being inactive) in Luxembourg canton (1.47 (0.98, 2.34)) and municipality (1.85 (1.06, 3.45)), whereas the lowest likelihood for being physical inactivity was noted in Vianden (0.64 (0.23, 1.20)) (Figure 2I; Tables S17 and S18).

## 4. Discussion

This report represents the first comprehensive snapshot of the situation in regard to the geographical variations in the top leading preventable and treatable cardio metabolic risk factors among the general population in Luxembourg.

Our study findings are important and will be useful for informing health policy makers on how to direct available resources toward cost-effective interventions by targeting at-risk communities. In view of the prominent burden of CVD, this research will help to better manage and fight against this escalating public health problem.

In general, higher concentration of smoking was observed in the northern regions, higher overweight/obesity and abdominal obesity were clustered in the central belt, whereas hypertension has been spotted in the southern part of the country. Noteworthy, our previous report, based on the ORISCAV-LUX dataset, documented remarkably high prevalence estimates of major potentially modifiable cardio metabolic risk factors among the adult residents in Luxembourg [8,17]. The present study additionally confirmed a heterogonous geographical distribution and risk pattern of these factors, thus pointing to geographic gradients and inequalities across the country. These extended findings provide added-value to our previous research on the epidemiology of cardio metabolic risk factors in Luxembourg.

Despite the small size of Luxembourg, canton- and municipality-specific rates did not always correspond with nationally reported rates; certain cantons/municipalities had a unique risk factor profile. Specifically, the Bayesian maps revealed that subjects residing in Luxembourg canton were significantly less likely to have hypertension or overweight/obesity, although they were less likely to be practicing physical activity of ≥8000 MET-min/week. These patterns were also observed at the level of Luxembourg municipality. Luxembourg is the capital and among the most populated sites of Luxembourg, inhabited by people from different cultures and origins. There are many facilities for cycling, fitness centres, but also easier access to public transport. It is possible that the people living in Luxembourg canton use public transport and practice office-based sedentary jobs. In addition to having lower global and abdominal obesity, the Luxembourg canton inhabitants would have a pretext for being less physically active, although further research is needed to prove this theory. In contrast to Luxembourg canton/municipality, the canton and municipality of Mersch were characterized by subjects with high abdominal obesity risk profiles.

The likelihood of alcohol use was remarkably low in Esch-sur-Alzette, a canton situated in the southwestern industrial area. Notably, an agglomeration of alcohol use was observed in Remich and Grevenmacher, wine-making dominated areas. Statistically, there were non-significant spatial patterns regarding smoking, diabetes, total serum cholesterol, and low eGFR risk distribution.

Our findings confirmed that when cantons and municipalities were analysed individually, there was a variability in risk factor profiles. Although not striking, these variances imply that geography-specific culture or spatial-related conditions might impact subjects’ behavioural and biological characteristics irrespective of their age and sex. Nevertheless, we should be cautious in the interpretation of the observed geographical variation due to the low number of participants in several relatively small municipalities.

Among a few similar studies, important geographical disparities in cardio metabolic risk factors have been reported in Europe [29] and worldwide [9,10,11,12,13,14]. To our knowledge, this is the first most comprehensive study to address the spatial effect of top leading behavioural and cardio metabolic risk factors in Luxembourg. Each risk factor had a different distribution among the 12 cantons and 106 municipalities over the country, suggesting the potential role of geographical factors. The present findings may provide evidence of the environmental role in the pathogenesis of CVD.

The reasons for the geographical differences in the patterns of selected risk factors are likely to be complex. One potential reason for geographic gradients in health is that they might simply reflect a concentration of people with lower socio-economic status, with their concordant health problems [29,30]. In a relatively homogenous country such as Luxembourg, urban versus rural areas are difficult to distinguish. Nevertheless, some of the variances between cantons and municipalities would be partially explained by the differences in the characteristics of individual areas within these regions. It would be plausible that people residing in Luxembourg municipality, classified as urban or more prosperous, would have better health status than those cantons/municipalities labelled as rural or relatively deprived. Additionally, the distribution of healthcare facilities is more favourable in the south and central part of the country, although regional resources are generally well allocated to meet the public’s health needs. Further research should focus on the characteristics of areas and the interplay between place and individuals.

Although we may not understand all the nuances that create risk factor variance, recognizing risk areas of a country serves to improve preventive initiatives so that resources are allocated only for necessary intervention (e.g., increase awareness for high abdominal obesity in Mersch and lower physical activity in Luxembourg). Consequently, these findings may help public health officials, medical professionals and health promoters to develop targeted prevention plans to narrow the gap and eliminate risk disparities for CVD in Luxembourg.

Noteworthy, the administrations of each municipality develop their own initiatives for health promotion of the inhabitants. It would be more appropriate to harmonize the efforts to bring improvements in health across the country and to reduce health inequalities.

There are several caveats to consider with this study. First, the cross-sectional design does not permit any causal inference regarding geographical location and the selected cardio metabolic risk factors, though reverse causation in this context is unlikely. Second, although remarkable between-municipalities and between-cantons variances exist, residual confounding related to socio-economic, dietary and lifestyle behaviours cannot be ruled out as a possible explanation for the observed findings. To confirm that the observed differences are independently related to geographic reasons, or, in other words, that the geographic inequalities are merely a result of differences in the socio-economic structure of the areas, further adjustments to these confounders are warranted.

Despite these limitations, our study expands the body of knowledge regarding a global vision of the geographical epidemiology of these cardio metabolic risk factors in Luxembourg, a small representative centrally-located European country. Using a nationwide representative sample of the adult population residing in the country, this study demonstrated remarkable geographic differences in the prevalence of the major behavioural and metabolic risk factors across cantons and municipalities. These findings are of particular interest at the European level, as they advance the hypothesis of European disparities in the distribution of cardio metabolic risk factors among other European countries. Our findings contribute to filling gaps on the worldwide map examining variations in geographical risk distribution.

Additionally, the strengths of the study reside first in the inclusion of a large nationally representative population-based sample of adults. Second, the comparison of known demographic and cardiovascular health-related profiles of the participants and non-participants to the ORISCAV-LUX survey was demonstrated earlier [19]; the participants did not differ substantially from the non-participants, and the response rate allowed the findings to be generalized for the entire population. Third, although the young age group was underrepresented in the sample, the data were weighted to provide population-representative prevalence estimates. Fourth, the ORISCAV-LUX survey provided reliable objective measurements, performed by well-trained personnel, of major cardio metabolic risk factors, namely, obesity, diabetes, hypertension, low eGFR and lipid disorders. This helps in avoiding the retrieval of these factors from relatively inaccurate self-administered information, though smoking, alcohol use and physical activity were based on self-reported data.

## 5. Conclusions

In conclusion, this study provides a national vision for the global burden of CVD in Luxembourg by presenting the geographical mapping of the top leading behavioural and metabolic risk factors. It shows that there is significant variation in the distribution of several preventable and treatable risk factors, in particular hypertension, overweight/obesity, and physical inactivity among the cantons and municipalities of Luxembourg. Along with the rising burden of these risk factors and associated pathologies, our findings have practical implications for healthcare practitioners and policy makers. Further prevention measures at specific canton/municipality levels should be considered to address the observed geographic inequalities.

## Figures and Tables

**Table 1:** Definitions of top leading behavioural and metabolic risk factors [3].

Top Leading Risk Factors	Definition
Smoking	Current daily or occasional tobacco consumption
High BMI (overweight/obesity)	Body-mass index > 25 kg/m^2^ were considered as overweight/obese [23]
High BP (hypertension)	SBP ≥ 140 mmHg and/or DBP ≥ 90 mmHg and/or antihypertensive medications intake [24]
High FPG (diabetes)	FPG ≥ 126 mg/dL and/or antidiabetic medications intake [25]
Alcohol use	Daily alcohol consumption measured in mL per day
High total cholesterol	Total cholesterol >4.8 mmol/L (185.6 mg/dL) [3]
Low GFR (CKD) *	eGFR <60 mL per min per 1.73 m^2^ [26]
Low physical activity	Weekly physical activity <8000 Metabolic Equivalent of Task (MET) min per week [3]
High abdominal obesity	Waist circumference (WC) ≥ 102 cm for men and ≥88 cm for women [27]

* Estimated glomerular filtration rate (eGFR) = 175 × (Creatinine) − 1.154 × (Age) − 0.203 × (0.742 if female) × (1.212 if black) measured in mL/min/1.73 m^2^, to indicate chronic kidney disease (CKD). BMI, body mass index; BP, blood pressure; SBP, systolic blood pressure; DBP, diastolic blood pressure; FPG, fasting plasma glucose; GFR, glomerular filtration rate.

**Table 2:** Prevalence of the top leading behavioural and metabolic risk factors according to sex among participants in ORISCAV-LUX, 2007–2008 (*N* = 1430).

Overall Characteristics	*N* *	Total Sample	Men	Women	*p*-Value
Total n (%)			696 (50.38)	734 (49.62)	
Age, years		42.03 (0.04)	41.89 (0.06)	42.16 (0.06)	0.66
Tobacco Consumption, (%)	1430				0.04
Non-smoker		1123 (77.69)	531(75.10)	592 (80.32)	
Smoker		307 (22.31)	165 (24.90)	142 (19.68)	
Body Mass Index, (%)	1429				<0.001
Low BMI		621 (46.11)	227 (36.09)	394 (56.27)	
High BMI		808 (53.89)	468 (63.91)	340 (43.73)	
Abdominal obesity, (%)	1429				<0.001
Non-obese		968 (70.14)	505 (74.89)	463 (65.33)	
Obese		461 (29.86)	190 (25.11)	271 (34.67)	
Hypertension, (%)	1429				<0.001
Non-hypertensive		889 (65.42)	373 (58.04)	516 (72.91)	
Hypertensive		540 (34.58)	322 (41.96)	218 (27.09)	
Diabetes, (%)	1396				0.17
Non-diabetic		1327 (95.64)	638 (94.82)	689 (96.46)	
Diabetic		69 (4.36)	39 (5.18)	30 (3.54)	
Alcohol Consumption, (%)	1350				<0.001
Non-drinker		229 (17.53)	68 (10.91)	161 (24.25)	
Drinker		1121 (82.47)	588 (89.09)	533 (75.75)	
Total Cholesterol, (%)	1425				0.67
Low TC		519 (39.78)	257 (40.35)	262 (39.20)	
High TC		906 (60.22)	438 (59.65)	468 (60.80)	
Low eGFR, (%)	1425				0.59
≥60		1401 (98.51)	682 (98.35)	719 (98.67)	
<60		24 (1.49)	13 (1.65)	11 (1.33)	
Physical Activity, (%)	1364				0.65
Low physical activity		1225 (89.48)	592 (88.70)	633 (90.27)	
High physical activity		139 (10.52)	70 (11.30)	69 (9.73)	

*p*-values were calculated by using χ^2^ test and Mann-Whitney U-test for categorical and continuous variables, which were presented as number (proportions in %) and means ± Standard Error, respectively. * Differences in sample sizes are due to missing data.

## Supplementary Materials

The following are available online at www.mdpi.com/1660-4601/14/6/648/s1, Tables S1–S18: Results of descriptive, multivariable logistic. Bayesian regression analyses (based on MCMC technique) for the nine selected leading behavioural and metabolic risk factors.
