# Supplementary material for: Geographic Variations in Cardiometabolic Risk Factors in Luxembourg
Source: Int J Environ Res Public Health. 2017 Jun 16;14(6):648. doi: 10.3390/ijerph14060648 (PMC5486334; doi:10.3390/ijerph14060648)
Supplement: Supplementary file 1 [file ijerph-14-00648-s001.pdf]

International Journal of
Environmental Research and Public Health

MDPI

Geographic Variations in Cardiometabolic Risk Factors in Luxembourg

Ala'a Alkerwi, Illiasse El Bahi, Saverio Stranges, Jean Beissel, Charles Delagardelle, Stephanie Noppe and Ngianga-Bakwin Kandala

Supplementary Materials

Table S1. Smoking according to cantons.

	Crude prevalence N (%)	Age- and sex-adjusted OR (95% CI)	Age- and sex-adjusted POR (95% CI)
Age, mean (SE), years	38.3 (0.66)	0.98 (0.97, 0.99)	0.98 (0.97, 0.99)
Sex			
Male	165 (24.90)	1.00	1.00
Female	142 (19.68)	0.78 (0.60, 1.01)	0.77 (0.61, 0.99)
Canton †			
LUXEMBOURG	72 (19.01)	1.00	0.89 (0.68, 1.09)
CLERVAUX	16 (38.10)	2.79 (1.41, 5.55)	1.19 (0.88, 1.90)
DIEKIRCH	16 (19.60)	1.04 (0.57, 1.92)	0.97 (0.71, 1.23)
REDANGE	5 (15.74)	0.73 (0.27, 1.94)	0.93 (0.61, 1.25)
VIANDEN	1 (10.11)	0.48 (0.06, 3.86)	0.98 (0.55, 1.48)
WILTZ	12 (26.29)	1.68 (0.82, 3.45)	1.08 (0.80, 1.60)
ECHTERNACH	11 (27.30)	1.45 (0.69, 3.02)	1.02 (0.74, 1.40)
GREVENMACHER	15 (20.47)	1.20 (0.64, 2.25)	0.97 (0.72, 1.29)
REMICH	13 (25.00)	1.41 (0.71, 2.80)	1.00 (0.71, 1.40)
CAPELLEN	26 (21.13)	1.15 (0.69, 1.90)	0.96 (0.72, 1.24)
ESCH/ALZETTE	100 (23.85)	1.37 (0.97, 1.93)	1.04 (0.83, 1.33)
MERSCH	20 (25.55)	1.34 (0.76, 2.35)	1.00 (0.76, 1.30)

†p = 0.19.

Table S2. Smoking according to municipalities.

	Crude prevalence N (%)	Age- and sex-adjusted OR (95% CI)	Age- and sex-adjusted POR (95% CI)
Municipality †			
LUXEMBOURG	37 (18.98)	1.00	0.92 (0.69, 1.13)
BEAUFORT	3 (76.79)	10.42 (1.05, 103.53)	1.05 (0.76, 1.54)
BECH	0 (0.00)	0.00 (0.00, Infinity)	0.97 (0.69, 1.30)
BECKERICH	0 (0.00)	0.00 (0.00, Infinity)	0.95 (0.63, 1.27)
BERDORF	1 (18.72)	0.82 (0.09, 7.43)	0.99 (0.69, 1.41)
BERTRANGE	6 (19.84)	1.13 (0.43, 2.97)	0.97 (0.71, 1.28)
BETTEMBOURG	4 (12.68)	0.76 (0.25, 2.33)	0.97 (0.70, 1.26)
BETTENDORF	3 (29.60)	2.38 (0.58, 9.79)	1.03 (0.75, 1.52)
BETZDORF	1 (19.32)	1.17 (0.13, 10.53)	0.98 (0.71, 1.29)
BISSEN	3 (69.37)	6.00 (0.95, 38.09)	1.03 (0.76, 1.45)
BIWER	1 (27.28)	0.99 (0.11, 9.02)	0.97 (0.70, 1.33)
BOEVANGE/ATTERT	1 (21.48)	1.51 (0.15, 15.11)	1.00 (0.72, 1.39)
BOURSCHEID	0 (0.00)	0.00 (0.00, Infinity)	1.02 (0.76, 1.42)
BOUS	0 (0.00)	0.00 (0.00, Infinity)	0.96 (0.66, 1.35)
CLERVAUX	5 (33.14)	2.60 (0.81, 8.33)	1.12 (0.80, 1.79)
COLMAR-BERG	2 (22.22)	1.00 (0.20, 5.01)	0.98 (0.70, 1.34)

CONSDORF	1 (20.47)	1.35 (0.14, 12.73)	0.99 (0.71, 1.39)
CONTERN	0 (0.00)	0.00 (0.00, Infinity)	0.93 (0.66, 1.22)
DALHEIM	2 (24.83)	1.45 (0.28, 7.58)	0.98 (0.73, 1.37)
DIEKIRCH	3 (18.64)	0.95 (0.26, 3.49)	0.99 (0.73, 1.37)
DIFFERDANGE	9 (20.24)	1.20 (0.52, 2.75)	1.02 (0.74, 1.42)
DIPPACH	4 (32.92)	1.58 (0.47, 5.33)	1.01 (0.75, 1.42)
DUDELANGE	16 (27.42)	1.76 (0.89, 3.48)	1.07 (0.84, 1.51)
ECHTERNACH	3 (27.61)	1.24 (0.32, 4.70)	0.98 (0.70, 1.34)
ELL	0 (0.00)	0.00 (0.00, Infinity)	0.99 (0.64, 1.45)
ERPELDANGE	1 (13.81)	1.30 (0.13, 13.20)	1.00 (0.72, 1.40)
ESCH/ALZETTE	20 (23.77)	1.49 (0.80, 2.78)	1.06 (0.84, 1.45)
ESCH/SURE	2 (30.07)	1.64 (0.30, 8.87)	1.02 (0.74, 1.39)
ETTELBRUCK	3 (14.35)	0.64 (0.18, 2.28)	0.97 (0.71, 1.28)
FEULEN	1 (57.57)	6.57 (0.40, 108.15)	1.03 (0.74, 1.46)
FISCHBACH	1 (35.35)	1.62 (0.16, 16.12)	0.99 (0.71, 1.33)
FLAXWEILER	2 (38.62)	5.62 (0.75, 41.89)	1.01 (0.73, 1.43)
FRISANGE	4 (29.72)	1.35 (0.41, 4.40)	1.00 (0.70, 1.40)
GARNICH	1 (19.47)	1.37 (0.15, 12.76)	1.00 (0.70, 1.43)
GOESDORF	3 (43.76)	2.81 (0.64, 12.45)	1.07 (0.81, 1.58)
GREVENMACHER	4 (19.04)	1.20 (0.37, 3.86)	0.97 (0.70, 1.32)
GROSBOUS	0 (0.00)	0.00 (0.00, Infinity)	0.99 (0.69, 1.34)
HEFFINGEN	0 (0.00)	0.00 (0.00, Infinity)	0.98 (0.70, 1.32)
HESPERANGE	8 (19.13)	1.23 (0.52, 2.90)	0.98 (0.72, 1.31)
HOBSCHEID	2 (17.95)	1.12 (0.23, 5.46)	0.97 (0.69, 1.32)
JUNGLINSTER	1 (7.12)	0.30 (0.04, 2.36)	0.93 (0.66, 1.18)
KAERJEN	11 (39.41)	2.90 (1.26, 6.67)	1.10 (0.86, 1.56)
KAYL	9 (28.74)	1.60 (0.68, 3.73)	1.05 (0.78, 1.48)
KEHLEN	3 (22.27)	1.23 (0.33, 4.63)	0.97 (0.68, 1.27)
KIISCHPELT	0 (0.00)	0.00 (0.00, Infinity)	1.06 (0.76, 1.65)
KOERICH	0 (0.00)	0.00 (0.00, Infinity)	0.92 (0.60, 1.19)
KOPSTAL	2 (17.24)	1.36 (0.28, 6.68)	0.97 (0.70, 1.31)
LAC DE LA HAUTE SURE	2 (18.21)	2.47 (0.43,14.17)	1.06 (0.78, 1.53)
LAROCHETTE	5 (43.10)	3.11 (0.92,10.55)	1.03 (0.78, 1.53)
LENNINGEN	4 (40.44)	3.30 (0.84,13.01)	1.01 (0.74, 1.47)
LEUDELANGE	1 (26.49)	2.06 (0.18,23.86)	1.00 (0.75, 1.39)
LINTGEN	1 (9.72)	0.64 (0.08,5.28)	0.95 (0.63, 1.32)
LORENTZWEILER	1 (15.80)	0.72 (0.09,6.02)	0.95 (0.64, 1.23)
MAMER	2 (9.90)	0.47 (0.10,2.12)	0.94 (0.66, 1.22)
MANTERNACH	1 (30.56)	1.72 (0.15,19.74)	0.97 (0.68, 1.35)
MERSCH	3 (11.44)	0.57 (0.16,2.01)	0.95 (0.69, 1.19)
MERTERT	3 (21.64)	1.36 (0.36,5.17)	0.98 (0.68, 1.41)
MERTZIG	2 (66.67)	4.03 (0.54,30.28)	1.03 (0.74, 1.45)
MOMPACH	0 (0.00)	0.00 (0.00,Infinity)	0.96 (0.65, 1.32)
MONDERCANGE	4 (21.64)	1.32 (0.41,4.26)	1.01 (0.76, 1.39)
MONDORF-LES-BAINS	3 (27.97)	1.57 (0.40,6.20)	0.99 (0.66, 1.47)
NIEDERANVEN	3 (17.20)	0.75 (0.21,2.70)	0.94 (0.65, 1.25)
NOMMERN	1 (25.97)	2.04 (0.17,24.22)	1.00 (0.75, 1.36)
PARC HOSINGEN	5 (52.77)	4.51 (1.21,16.78)	1.11 (0.83, 1.81)
PETANGE	11 (31.15)	1.69 (0.76,3.73)	1.07 (0.75, 1.62)
PREIZERDAUL	1 (40.40)	5.34 (0.30,95.11)	1.01 (0.74, 1.43)
PUTSCHEID	0 (0.00)	0.00 (0.00,Infinity)	1.02 (0.70, 1.54)
RAMBROUCH	1 (28.89)	1.12 (0.13, 9.97)	1.00 (0.72, 1.35)
RECKANGE/MESS	1 (10.92)	0.38 (0.05, 3.03)	0.97 (0.69, 1.29)
REDANGE	2 (23.09)	1.35 (0.27, 6.87)	0.99 (0.69, 1.37)

REISDORF	0 (0.00)	0.00 (0.00, Infinity)	1.00 (0.71, 1.42)
REMICH	0 (0.00)	0.00 (0.00, Infinity)	0.94 (0.63, 1.32)
ROESER	3 (17.18)	0.83 (0.23, 3.01)	0.98 (0.73, 1.27)
ROSPORT	2 (19.15)	1.21 (0.24, 6.10)	0.97 (0.65, 1.37)
RUMELANGE	3 (27.20)	2.46 (0.58, 10.54)	1.06 (0.76, 1.67)
SAEUL	0 (0.00)	0.00 (0.00, Infinity)	0.98 (0.69, 1.34)
SANDWEILER	2 (13.80)	0.87 (0.18, 4.23)	0.96 (0.71, 1.25)
SANEM	11 (24.47)	1.63 (0.75, 3.55)	1.04 (0.80, 1.42)
SCHENGEN	3 (20.92)	1.29 (0.33, 4.99)	0.98 (0.67, 1.37)
SCHIEREN	0 (0.00)	0.00 (0.00, Infinity)	0.98 (0.71, 1.33)
SCHIFFLANGE	4 (22.12)	1.28 (0.40, 4.13)	1.02 (0.73, 1.49)
SCHUTTRANGE	1 (12.28)	0.41 (0.05, 3.36)	0.94 (0.65, 1.24)
STADTBREDIMUS	1 (100.00)	106523681.21 (0.00, Infinity)	0.98 (0.69, 1.37)
STEINFORT	1 (7.88)	0.41 (0.05, 3.34)	0.96 (0.67, 1.24)
STEINSEL	1 (13.80)	0.44 (0.05, 3.52)	0.94 (0.67, 1.21)
STRASSEN	4 (35.98)	1.95 (0.56, 6.76)	1.00 (0.76, 1.35)
TANDEL	1 (17.21)	0.80 (0.09, 6.94)	1.01 (0.76, 1.41)
TROISVIERGES	5 (47.76)	3.60 (1.02, 12.71)	1.15 (0.78, 2.16)
TUNTANGE	2 (100.00)	182977271.24 (0.00, Infinity)	1.01 (0.72, 1.46)
USELDANGE	1 (18.81)	1.23 (0.13, 11.39)	0.99 (0.71, 1.32)
VALLéE DE L'ERNZ	3 (22.00)	1.29 (0.33, 5.06)	1.01 (0.77, 1.41)
VIANDEN	0 (0.00)	0.00 (0.00, Infinity)	1.01 (0.68, 1.53)
WAHL	0 (0.00)	0.00 (0.00, Infinity)	1.00 (0.69, 1.41)
WALDBILLIG	1 (51.92)	5.21 (0.29, 95.31)	1.01 (0.72, 1.43)
WALDBREDIMUS	0 (0.00)	0.00 (0.00, Infinity)	0.97 (0.70, 1.33)
WALFERDANGE	8 (23.96)	1.62 (0.67, 3.94)	1.00 (0.71, 1.38)
WEILER-LA-TOUR	2 (49.94)	4.62 (0.63, 33.90)	1.00 (0.73, 1.40)
WEISWAMPACH	0 (0.00)	0.00 (0.00, Infinity)	1.10 (0.73, 1.95)
WILTZ	5 (27.26)	1.80 (0.60, 5.44)	1.07 (0.80, 1.60)
WINCRANGE	1 (11.57)	1.03 (0.11, 9.81)	1.08 (0.76, 1.73)
WINSELER	0 (0.00)	0.00 (0.00, Infinity)	1.05 (0.72, 1.60)
WORMELDANGE	2 (37.02)	3.27 (0.52, 20.78)	1.00 (0.70, 1.44)

† $p = 0.26$.

Table S3. High body mass index (overweight/obesity) according to cantons.

	**Crude prevalence N (%)**	**Age- and sex-adjusted OR (95% CI)**	**Age- and sex-adjusted POR (95% CI)**
Age, mean (SE), years	46.2 (0.34)	1.05 (1.04, 1.06)	1.05 (1.04, 1.06)
Sex			
Male	468 (63.91)	1.00	1.00
Female	340 (43.73)	0.38 (0.30, 0.47)	0.37 (0.29, 0.46)
Canton†			
LUXEMBOURG	201 (49.22)	1.00	0.84 (0.64, 1.03)
CLERVAUX	19 (44.86)	0.88 (0.43, 1.79)	0.94 (0.63, 1.26)
DIEKIRCH	49 (53.04)	1.50 (0.90, 2.52)	1.04 (0.81, 1.38)
REDANGE	21 (54.37)	1.27 (0.62, 2.58)	1.01 (0.75, 1.37)
VIANDEN	7 (64.67)	1.30 (0.36, 4.70)	1.01 (0.70, 1.53)
WILTZ	31 (61.89)	2.16 (1.05, 4.42)	1.11 (0.84, 1.61)
ECHTERNACH	29 (65.11)	1.94 (0.95, 3.93)	1.08 (0.81, 1.55)
GREVENMACHER	38 (50.54)	1.06 (0.62, 1.83)	0.94 (0.68, 1.20)

REMICH	31 (54.46)	1.44 (0.78, 2.67)	1.00 (0.72, 1.37)
CAPELLEN	76 (55.60)	1.27 (0.83, 1.95)	0.97 (0.75, 1.27)
ESCH/ALZETTE	249 (55.31)	1.42 (1.05, 1.91)	1.04 (0.86, 1.30)
MERSCH	57 (61.81)	1.75 (1.04, 2.93)	1.07 (0.83, 1.42)

† $p = 0.16$.

Table S4. High body mass index (overweight/obesity) according to municipalities.

	**Crude prevalence N (%)**	**Age- and sex-adjusted OR (95% CI)**	**Age- and sex-adjusted POR (95% CI)**
Municipality †			
LUXEMBOURG	102 (47.67)	1.00	0.85 (0.62, 1.08)
BEAUFORT	4 (100.00)	26796044.67 (0.00, Infinity)	1.12 (0.70, 1.95)
BECH	1 (100.00)	6621982.46 (0.00, Infinity)	1.00 (0.61, 1.75)
BECKERICH	5 (62.35)	2.04 (0.43, 9.58)	1.04 (0.64, 1.76)
BERDORF	4 (69.98)	1.48 (0.24, 9.12)	1.03 (0.64, 1.67)
BERTRANGE	16 (51.50)	0.95 (0.42, 2.14)	0.92 (0.59, 1.29)
BETTEMBOURG	17 (55.05)	1.54 (0.67, 3.55)	1.03 (0.71, 1.56)
BETTENDORF	6 (62.15)	0.84 (0.21, 3.42)	0.99 (0.58, 1.55)
BETZDORF	3 (48.33)	0.80 (0.14, 4.69)	0.94 (0.58, 1.53)
BISSEN	4 (70.33)	5.31 (0.56, 50.34)	1.10 (0.73, 1.82)
BIWER	2 (32.04)	0.23 (0.03, 1.58)	0.90 (0.53, 1.36)
BOEVANGE/ATTERT	2 (44.18)	0.93 (0.10, 8.46)	1.02 (0.62, 1.64)
BOURSCHEID	2 (100.00)	20820434.82 (0.00, Infinity)	1.06 (0.65, 1.79)
BOUS	2 (100.00)	15551681.83 (0.00, Infinity)	1.06 (0.67, 1.84)
CLERVAUX	4 (24.65)	0.44 (0.12, 1.60)	0.88 (0.49, 1.32)
COLMAR-BERG	4 (30.43)	0.56 (0.14, 2.28)	0.96 (0.58, 1.50)
CONSDORF	3 (60.67)	1.09 (0.17, 6.89)	0.99 (0.61, 1.63)
CONTERN	9 (67.08)	2.77 (0.73, 10.51)	1.04 (0.69, 1.73)
DALHEIM	3 (29.07)	0.73 (0.16, 3.39)	0.95 (0.58, 1.45)
DIEKIRCH	8 (39.25)	0.91 (0.32, 2.62)	0.95 (0.59, 1.45)
DIFFERDANGE	27 (61.17)	2.27 (1.06, 4.84)	1.17 (0.83, 1.95)
DIPPACH	10 (60.31)	2.12 (0.65, 6.93)	1.04 (0.67, 1.63)
DUDELANGE	35 (55.17)	1.11 (0.60, 2.06)	0.95 (0.65, 1.32)
ECHTERNACH	6 (43.25)	0.50 (0.15, 1.66)	0.90 (0.53, 1.36)
ELL	1 (100.00)	9908106.45 (0.00, Infinity)	1.03 (0.60, 1.86)
ERPELDANGE	2 (41.62)	1.32 (0.17, 10.37)	1.01 (0.62, 1.60)
ESCH/ALZETTE	44 (54.53)	1.19 (0.68, 2.10)	0.98 (0.68, 1.37)
ESCH/SURE	5 (68.44)	4.18 (0.68, 25.64)	1.11 (0.75, 1.83)
ETTELBRUCK	17 (61.58)	2.44 (0.91, 6.56)	1.14 (0.77, 1.91)
FEULEN	1 (57.57)	0.42 (0.02, 8.08)	1.02 (0.63, 1.65)
FISCHBACH	2 (43.17)	1.29 (0.15, 11.04)	1.00 (0.63, 1.62)
FLAXWEILER	3 (71.37)	2.91 (0.28, 30.70)	1.00 (0.60, 1.71)
FRISANGE	10 (59.16)	1.53 (0.51, 4.63)	1.01 (0.66, 1.64)
GARNICH	2 (42.23)	0.46 (0.06, 3.45)	0.95 (0.53, 1.53)
GOESDORF	7 (91.60)	6.75 (0.80, 56.87)	1.14 (0.78, 2.03)
GREVENMACHER	9 (43.51)	1.03 (0.36, 2.95)	0.95 (0.59, 1.46)
GROSBOUS	3 (100.00)	31057448.60 (0.00, Infinity)	1.13 (0.70, 2.00)
HEFFINGEN	0 (0.00)	0.00 (0.00, Infinity)	0.97 (0.59, 1.59)
HESPERANGE	18 (46.00)	0.67 (0.31, 1.43)	0.83 (0.50, 1.17)
HOBSCHEID	6 (55.89)	0.85 (0.23, 3.14)	0.95 (0.58, 1.52)
JUNGLINSTER	7 (47.66)	0.64 (0.21, 1.91)	0.89 (0.54, 1.31)
KAERJEN	20 (65.90)	1.73 (0.72, 4.17)	1.05 (0.73, 1.59)

KAYL	20 (57.25)	1.35 (0.60, 3.04)	1.00 (0.65, 1.53)
KEHLEN	9 (60.48)	1.32 (0.41, 4.22)	0.99 (0.65, 1.51)
KIISCHPELT	0 (0.00)	0.00 (0.00, Infinity)	0.98 (0.56, 1.56)
KOERICH	9 (71.65)	2.16 (0.61, 7.63)	1.05 (0.71, 1.72)
KOPSTAL	7 (62.79)	1.05 (0.28, 3.89)	0.95 (0.60, 1.50)
LAC DE LA HAUTE SURE	4 (53.58)	1.91 (0.29, 12.73)	1.05 (0.66, 1.75)
LAROCHETTE	10 (84.09)	4.92 (0.96, 25.04)	1.15 (0.77, 2.08)
LENNINGEN	4 (44.07)	0.65 (0.16, 2.57)	0.92 (0.57, 1.39)
LEUDELANGE	3 (100.00)	14774394.90 (0.00, Infinity)	1.05 (0.64, 1.92)
LINTGEN	7 (70.23)	1.30 (0.31, 5.43)	1.00 (0.60, 1.67)
LORENTZWEILER	4 (46.76)	0.60 (0.14, 2.63)	0.93 (0.55, 1.39)
MAMER	11 (45.03)	0.99 (0.36, 2.73)	0.94 (0.61, 1.37)
MANTERNACH	2 (65.29)	1.79 (0.15, 20.87)	0.99 (0.60, 1.61)
MERSCH	21 (73.04)	3.30 (1.24, 8.82)	1.18 (0.85, 2.12)
MERTERT	9 (60.59)	2.08 (0.59, 7.34)	1.04 (0.63, 1.66)
MERTZIG	4 (100.00)	14895232.85 (0.00, Infinity)	1.12 (0.76, 1.98)
MOMPACH	1 (38.50)	0.34 (0.02, 5.43)	0.95 (0.52, 1.51)
MONDERCANGE	11 (48.69)	0.93 (0.34, 2.55)	0.96 (0.63, 1.42)
MONDORF-LES-BAINS	7 (56.11)	1.33 (0.36, 4.87)	1.00 (0.59, 1.67)
NIEDERANVEN	12 (53.65)	0.92 (0.35, 2.39)	0.90 (0.56, 1.32)
NOMMERN	2 (51.93)	1.27 (0.09, 18.69)	1.03 (0.65, 1.63)
PARC HOSINGEN	8 (80.04)	2.81 (0.53, 14.98)	1.07 (0.70, 1.72)
PETANGE	14 (33.85)	0.59 (0.27, 1.28)	0.79 (0.43, 1.18)
PREIZERDAUL	0 (0.00)	0.00 (0.00, Infinity)	0.96 (0.55, 1.54)
PUTSCHEID	2 (100.00)	10891667.22 (0.00, Infinity)	1.07 (0.68, 1.82)
RAMBROUCH	3 (55.31)	0.61 (0.11, 3.40)	0.98 (0.59, 1.56)
RECKANGE/MESS	5 (36.70)	0.79 (0.22, 2.86)	0.94 (0.57, 1.47)
REDANGE	4 (41.60)	0.88 (0.21, 3.73)	0.99 (0.55, 1.53)
REISDORF	1 (35.03)	1.03 (0.09, 11.97)	1.00 (0.61, 1.62)
REMICH	5 (87.94)	4.55 (0.47, 43.61)	1.07 (0.67, 1.97)
ROESER	12 (54.92)	1.32 (0.47, 3.68)	0.98 (0.64, 1.58)
ROSPORT	8 (82.78)	17.05 (1.92, 151.50)	1.19 (0.80, 2.19)
RUMELANGE	6 (67.12)	3.32 (0.71, 15.46)	1.10 (0.72, 1.91)
SAEUL	1 (100.00)	46342984.36 (0.00, Infinity)	1.04 (0.64, 1.70)
SANDWEILER	6 (44.00)	0.77 (0.22, 2.71)	0.92 (0.57, 1.38)
SANEM	33 (70.79)	3.39 (1.52, 7.55)	1.27 (0.89, 2.22)
SCHENGEN	8 (58.07)	2.13 (0.63, 7.13)	1.06 (0.67, 1.91)
SCHIEREN	3 (58.23)	3.33 (0.22, 50.72)	1.07 (0.67, 1.80)
SCHIFFLANGE	12 (58.92)	1.73 (0.60, 4.97)	1.05 (0.67, 1.71)
SCHUTTRANGE	3 (27.14)	0.48 (0.11, 2.07)	0.88 (0.50, 1.29)
STADTBREDIMUS	0 (0.00)	0.00 (0.00, Infinity)	0.97 (0.58, 1.59)
STEINFORT	2 (19.65)	0.38 (0.07, 1.95)	0.89 (0.47, 1.30)
STEINSEL	8 (58.17)	1.17 (0.34, 3.99)	0.96 (0.62, 1.47)
STRASSEN	6 (43.70)	0.57 (0.17, 1.95)	0.88 (0.50, 1.35)
TANDEL	4 (52.69)	1.03 (0.21, 5.10)	1.00 (0.61, 1.55)
TROISVIERGES	4 (39.62)	0.66 (0.16, 2.71)	0.91 (0.47, 1.51)
TUNTANGE	1 (68.02)	1.33 (0.08, 22.56)	1.01 (0.60, 1.64)
USELDANGE	2 (39.94)	0.60 (0.09, 4.22)	0.97 (0.58, 1.51)
VALLéE DE L'ERNZ	5 (29.43)	0.85 (0.25, 2.92)	0.98 (0.61, 1.42)
VIANDEN	1 (66.49)	0.49 (0.03, 7.97)	0.99 (0.55, 1.71)
WAHL	2 (77.12)	1.29 (0.10, 16.12)	1.04 (0.60, 1.86)
WALDBILLIG	2 (100.00)	12060029.70 (0.00, Infinity)	1.05 (0.62, 1.80)
WALDBREDIMUS	2 (100.00)	3371744.99 (0.00, Infinity)	1.01 (0.62, 1.76)
WALFERDANGE	18 (55.82)	1.24 (0.54, 2.86)	0.96 (0.63, 1.41)

WEILER-LA-TOUR	3 (75.71)	3.25 (0.30, 34.64)	1.01 (0.63, 1.68)
WEISWAMPACH	0 (0.00)	0.00 (0.00, Infinity)	0.96 (0.53, 1.65)
WILTZ	12 (51.43)	1.38 (0.47, 4.04)	1.03 (0.67, 1.65)
WINCRANGE	3 (51.41)	1.49 (0.20, 11.21)	1.00 (0.63, 1.71)
WINSELER	3 (79.15)	2.28 (0.19, 27.46)	1.04 (0.64, 1.75)
WORMELDANGE	3 (56.18)	2.00 (0.28, 14.13)	0.99 (0.60, 1.64)

† $p = 0.05$.

Table S5. Abdominal obesity according to cantons.

	**Crude prevalence N (%)**	**Age- and sex-adjusted OR (95% CI)**	**Age- and sex-adjusted POR (95% CI)**
Age, mean (SE), years	49.03 (0.53)	1.06 (1.05, 1.07)	1.06 (1.05 ,1.07)
Sex			
Male	190 (25.11)	1.00	1.00
Female	271 (34.67)	1.61 (1.27, 2.04)	1.60 (1.26, 2.01)
Canton†			
LUXEMBOURG	99 (23.02)	1.00	0.73 (0.53, 0.96)
CLERVAUX	13 (31.80)	1.54 (0.73, 3.25)	1.01 (0.64, 1.55)
DIEKIRCH	30 (29.06)	1.85 (1.08, 3.14)	1.11 (0.80, 1.63)
REDANGE	17 (47.75)	2.31 (1.13, 4.71)	1.16 (0.79, 1.86)
VIANDEN	3 (29.03)	0.91 (0.22, 3.77)	0.94 (0.49, 1.58)
WILTZ	16 (31.81)	1.83 (0.92, 3.63)	1.08 (0.74, 1.62)
ECHTERNACH	12 (25.31)	1.21 (0.57, 2.53)	0.94 (0.59, 1.36)
GREVENMACHER	22 (30.60)	1.19 (0.67, 2.13)	0.88 (0.59, 1.25)
REMICH	18 (32.25)	1.72 (0.90, 3.28)	1.02 (0.70, 1.52)
CAPELLEN	44 (31.14)	1.39 (0.89, 2.19)	0.94 (0.71, 1.27)
ESCH/ALZETTE	146 (31.49)	1.57 (1.14, 2.16)	1.01 (0.78, 1.31)
MERSCH	41 (42.27)	2.71 (1.62, 4.52)	1.30 (0.93, 2.06)

† $p = 0.02$.

Table S6. Abdominal obesity according to municipalities.

	**Crude prevalence N (%)**	**Age- and sex-adjusted OR (95% CI)**	**Age- and sex-adjusted POR (95% CI)**
Municipality †			
LUXEMBOURG	48 (21.50)	1.00	0.75 (0.53, 0.99)
BEAUFORT	0 (0.00)	0.00 (0.00, Infinity)	0.97 (0.53, 1.61)
BECH	0 (0.00)	0.00 (0.00, Infinity)	0.96 (0.57, 1.54)
BECKERICH	4 (47.65)	3.05 (0.71, 13.05)	1.13 (0.71, 1.96)
BERDORF	2 (30.12)	1.94 (0.30, 12.65)	0.99 (0.57, 1.71)
BERTRANGE	8 (25.34)	1.09 (0.44, 2.71)	0.88 (0.55, 1.29)
BETTEMBOURG	13 (43.81)	2.59 (1.09, 6.14)	1.09 (0.73, 1.79)
BETTENDORF	3 (28.75)	0.93 (0.22, 3.94)	0.98 (0.59, 1.65)
BETZDORF	3 (48.33)	1.94 (0.32, 11.59)	0.95 (0.59, 1.59)
BISSEN	3 (45.62)	8.95 (1.24, 64.50)	1.15 (0.73, 2.04)
BIWER	2 (29.29)	1.14 (0.17, 7.51)	0.94 (0.55, 1.50)
BOEVANGE/ATTERT	1 (22.82)	1.21 (0.12, 12.63)	1.04 (0.64, 1.72)
BOURSCHEID	1 (20.95)	5.40 (0.26, 110.22)	1.08 (0.64, 1.87)
BOUS	1 (43.32)	3.88 (0.22, 67.17)	0.98 (0.58, 1.71)
CLERVAUX	4 (24.29)	1.29 (0.36, 4.63)	0.98 (0.58, 1.57)
COLMAR-BERG	1 (6.74)	0.39 (0.05, 3.34)	0.98 (0.55, 1.52)
CONSDORF	2 (47.88)	1.50 (0.21, 10.75)	0.98 (0.59, 1.63)

CONTERN	5 (32.21)	2.06 (0.57, 7.48)	0.97 (0.59, 1.63)
DALHEIM	2 (20.12)	1.25 (0.22, 6.98)	0.94 (0.58, 1.49)
DIEKIRCH	6 (29.38)	2.04 (0.68, 6.15)	1.06 (0.65, 1.74)
DIFFERDANGE	14 (31.81)	2.04 (0.94, 4.41)	1.05 (0.69, 1.66)
DIPPACH	5 (31.63)	1.76 (0.52, 5.99)	0.98 (0.59, 1.55)
DUDELANGE	19 (28.62)	1.15 (0.59, 2.23)	0.88 (0.59, 1.27)
ECHTERNACH	1 (5.82)	0.22 (0.03, 1.84)	0.82 (0.40, 1.29)
ELL	0 (0.00)	0.00 (0.00, Infinity)	1.04 (0.61, 1.82)
ERPELDANGE	1 (13.81)	1.49 (0.12, 18.10)	1.03 (0.62, 1.69)
ESCH/ALZETTE	31 (35.42)	2.30 (1.27, 4.17)	1.16 (0.84, 1.76)
ESCH/SURE	2 (33.51)	1.92 (0.34, 10.75)	1.06 (0.67, 1.72)
ETTELBRUCK	12 (41.99)	3.11 (1.26, 7.68)	1.20 (0.81, 2.04)
FEULEN	0 (0.00)	0.00 (0.00, Infinity)	1.00 (0.56, 1.76)
FISCHBACH	2 (43.17)	3.41 (0.45, 25.78)	1.06 (0.67, 1.86)
FLAXWEILER	2 (51.87)	2.43 (0.31, 19.26)	0.97 (0.58, 1.67)
FRISANGE	4 (23.97)	1.18 (0.35, 3.93)	0.92 (0.55, 1.46)
GARNICH	1 (24.22)	0.57 (0.06, 5.51)	0.91 (0.50, 1.53)
GOESDORF	5 (62.20)	6.42 (1.38, 29.87)	1.19 (0.75, 2.22)
GREVENMACHER	5 (23.80)	1.19 (0.38, 3.73)	0.92 (0.54, 1.42)
GROSBOUS	3 (100.00)	59689836.16 (0.00, Infinity)	1.18 (0.73, 2.09)
HEFFINGEN	0 (0.00)	0.00 (0.00, Infinity)	1.00 (0.60, 1.65)
HESPERANGE	10 (24.01)	1.00 (0.43, 2.30)	0.84 (0.54, 1.22)
HOBSCHEID	5 (46.76)	2.47 (0.69, 8.86)	1.08 (0.66, 1.76)
JUNGLINSTER	3 (21.85)	0.70 (0.18, 2.69)	0.88 (0.52, 1.35)
KAERJEN	9 (28.58)	1.21 (0.50, 2.94)	0.92 (0.60, 1.36)
KAYL	9 (23.63)	1.31 (0.55, 3.12)	0.94 (0.58, 1.45)
KEHLEN	5 (29.88)	1.40 (0.43, 4.57)	0.97 (0.62, 1.46)
KIISCHPELT	0 (0.00)	0.00 (0.00, Infinity)	1.02 (0.57, 1.73)
KOERICH	8 (62.93)	5.72 (1.68, 19.50)	1.18 (0.78, 2.03)
KOPSTAL	4 (35.96)	0.96 (0.25, 3.67)	0.88 (0.51, 1.49)
LAC DE LA HAUTE SURE	2 (18.21)	1.51 (0.25, 9.02)	1.05 (0.62, 1.74)
LAROCHETTE	6 (44.51)	4.37 (1.22, 15.71)	1.18 (0.76, 2.08)
LENNINGEN	3 (32.79)	2.42 (0.56, 10.50)	0.97 (0.59, 1.64)
LEUDELANGE	1 (26.49)	2.27 (0.17, 30.44)	0.97 (0.59, 1.56)
LINTGEN	6 (60.11)	3.25 (0.82, 12.94)	1.12 (0.69, 1.92)
LORENTZWEILER	4 (46.76)	1.55 (0.37, 6.59)	1.02 (0.66, 1.61)
MAMER	6 (22.82)	1.08 (0.38, 3.10)	0.91 (0.56, 1.43)
MANTERNACH	0 (0.00)	0.00 (0.00, Infinity)	0.93 (0.51, 1.60)
MERSCH	16 (55.09)	4.45 (1.83, 10.81)	1.29 (0.89, 2.20)
MERTERT	6 (44.34)	2.22 (0.69, 7.19)	1.02 (0.62, 1.78)
MERTZIG	0 (0.00)	0.00 (0.00, Infinity)	1.02 (0.57, 1.73)
MOMPACH	0 (0.00)	0.00 (0.00, Infinity)	0.93 (0.54, 1.56)
MONDERCANGE	5 (21.87)	0.68 (0.22, 2.09)	0.86 (0.50, 1.31)
MONDORF-LES-BAINS	6 (48.62)	3.32 (0.93, 11.83)	1.09 (0.65, 2.02)
NIEDERANVEN	5 (19.39)	0.70 (0.23, 2.13)	0.84 (0.49, 1.26)
NOMMERN	2 (51.93)	11.02 (0.72, 167.98)	1.13(0.71, 1.95)
PARC HOSINGEN	5 (47.23)	4.05 (1.01, 16.24)	1.14 (0.69, 1.94)
PETANGE	7 (16.26)	0.89 (0.35, 2.25)	0.83 (0.51, 1.29)
PREIZERDAUL	1 (59.60)	2.71 (0.10, 72.86)	1.08 (0.65, 1.91)
PUTSCHEID	1 (40.41)	2.71 (0.11, 66.05)	1.06 (0.63, 1.77)
RAMBROUCH	3 (55.31)	2.30 (0.42, 12.61)	1.08 (0.68, 1.79)
RECKANGE/MESS	3 (22.39)	1.37 (0.34, 5.57)	0.94 (0.55, 1.50)
REDANGE	3 (33.93)	1.57 (0.35, 7.02)	1.05 (0.63, 1.70)

REISDORF	1 (35.03)	2.98 (0.22, 39.53)	1.03 (0.59, 1.73)
REMICH	2 (38.08)	1.40 (0.24, 8.20)	0.94 (0.51, 1.60)
ROESER	8 (35.48)	1.62 (0.59, 4.44)	0.95 (0.61, 1.44)
ROSPORT	6 (60.98)	10.00 (2.25, 44.46)	1.19 (0.72, 2.28)
RUMELANGE	4 (49.37)	2.49 (0.57, 10.89)	1.03 (0.60, 1.78)
SAEUL	1 (100.00)	88356534.32 (0.00, Infinity)	1.09 (0.67, 1.98)
SANDWEILER	3 (21.98)	1.05 (0.24, 4.50)	0.88 (0.53, 1.42)
SANEM	19 (40.08)	2.33 (1.13, 4.79)	1.11 (0.76, 1.73)
SCHENGEN	4 (30.29)	1.65 (0.45, 6.12)	0.96 (0.56, 1.60)
SCHIEREN	2 (36.62)	2.87 (0.30, 27.51)	1.08 (0.64, 1.91)
SCHIFFLANGE	9 (43.80)	2.86 (1.01, 8.08)	1.14 (0.72, 1.91)
SCHUTTRANGE	3 (26.49)	1.64 (0.40, 6.74)	0.94 (0.60, 1.53)
STADTBREDIMUS	0 (0.00)	0.00 (0.00, Infinity)	0.95 (0.56, 1.59)
STEINFORT	1 (7.42)	0.57 (0.07, 4.90)	0.93 (0.56, 1.46)
STEINSEL	3 (21.59)	0.66 (0.17, 2.62)	0.85 (0.49, 1.30)
STRASSEN	4 (29.06)	1.72 (0.48, 6.18)	0.96 (0.61, 1.58)
TANDEL	1 (11.09)	0.49 (0.05, 4.34)	0.96 (0.56, 1.54)
TROISVIERGES	3 (32.75)	1.39 (0.32, 6.15)	0.96 (0.55, 1.69)
TUNTANGE	0 (0.00)	0.00 (0.00, Infinity)	1.00 (0.59, 1.62)
USELDANGE	1 (21.13)	0.76 (0.08, 7.28)	1.03 (0.60, 1.74)
VALLéE DE L'ERNZ	4 (20.07)	2.56 (0.68, 9.67)	1.08 (0.69, 1.81)
VIANDEN	1 (66.49)	1.89 (0.08, 42.30)	1.05 (0.55, 1.87)
WAHL	1 (46.07)	1.30 (0.10, 17.25)	1.07 (0.63, 1.91)
WALDBILLIG	1 (48.08)	2.88 (0.09, 91.62)	1.01 (0.61, 1.71)
WALDBREDIMUS	0 (0.00)	0.00 (0.00, Infinity)	0.91 (0.52, 1.52)
WALFERDANGE	7 (19.39)	0.90 (0.35, 2.32)	0.81 (0.47, 1.25)
WEILER-LA-TOUR	3 (75.71)	11.71 (1.18, 116.03)	1.05 (0.68, 1.94)
WEISWAMPACH	0 (0.00)	0.00 (0.00, Infinity)	0.98 (0.50, 1.75)
WILTZ	6 (25.52)	1.57 (0.52, 4.72)	1.04 (0.62, 1.70)
WINCRANGE	1 (26.49)	0.77 (0.06, 9.30)	0.98 (0.53, 1.71)
WINSELER	1 (34.76)	1.01 (0.09, 11.03)	1.01 (0.57, 1.71)
WORMELDANGE	1 (26.82)	0.69 (0.07, 6.91)	0.91 (0.53, 1.55)

† $p = 0.13$.

Table S7. High blood pressure (hypertension) according to cantons.

	Crude prevalence N (%)	Age- and sex-adjusted OR (95% CI)	Age- and sex-adjusted POR (95% CI)
Age, mean (SE), years	51.01 (0.45)	1.10(1.08,1.11)	1.10(1.08,1.11)
Sex			
Male	322 (41.96)	1.00	1.00
Female	218 (27.09)	0.38 (0.30, 0.49)	0.38 (0.30, 0.49)
Canton†			
LUXEMBOURG	127 (29.96)	1.00	0.84 (0.62, 1.04)
CLERVAUX	13 (27.60)	1.04 (0.46, 2.36)	0.95 (0.63, 1.32)
DIEKIRCH	29 (31.04)	1.23 (0.69, 2.19)	0.97 (0.72, 1.30)
REDANGE	13 (35.22)	1.14 (0.52, 2.51)	0.96 (0.67, 1.29)
VIANDEN	8 (66.02)	5.31 (1.27, 22.22)	1.12 (0.78, 1.92)
WILTZ	19 (37.71)	1.54 (0.75, 3.16)	1.01 (0.72, 1.43)
ECHTERNACH	19 (40.08)	1.72 (0.83, 3.56)	1.04 (0.76, 1.49)
GREVENMACHER	26 (31.98)	1.15 (0.63, 2.10)	0.94 (0.67, 1.21)
REMICH	22 (38.66)	1.88 (0.97, 3.65)	1.07 (0.79, 1.57)
CAPELLEN	54 (38.62)	1.45 (0.91, 2.32)	1.01 (0.79, 1.32)
ESCH/ALZETTE	172 (36.34)	1.56 (1.12, 2.18)	1.08 (0.87, 1.37)

MERSCH	38 (38.55)	1.55 (0.90, 2.67)	1.02 (0.77, 1.34)

† $p = 0.13$.

Table S8. High blood pressure (hypertension) according to municipalities.

	**Crude prevalence N (%)**	**Age- and sex-adjusted OR (95% CI)**	**Age- and sex-adjusted POR (95% CI)**
Municipality †			
LUXEMBOURG	61 (27.71)	1.00	0.86 (0.63, 1.09)
BEAUFORT	1 (26.46)	1.48 (0.13, 16.43)	1.00 (0.64, 1.48)
BECH	1 (100.00)	17562189.43 (0.00, Infinity)	1.03 (0.73, 1.54)
BECKERICH	1 (9.63)	0.32 (0.03, 2.95)	0.94 (0.60, 1.34)
BERDORF	1 (13.65)	0.27 (0.03, 2.82)	0.96 (0.61, 1.44)
BERTRANGE	14 (42.64)	2.04 (0.86, 4.84)	1.04 (0.78, 1.52)
BETTEMBOURG	11 (34.19)	1.63 (0.64, 4.10)	1.04 (0.73, 1.53)
BETTENDORF	4 (47.50)	0.66 (0.14, 3.00)	0.98 (0.62, 1.50)
BETZDORF	2 (32.59)	0.62 (0.09, 4.18)	0.95 (0.61, 1.36)
BISSEN	1 (15.08)	0.93 (0.08, 10.30)	0.98 (0.64, 1.40)
BIWER	3 (47.09)	1.32 (0.16, 10.61)	0.99 (0.66, 1.46)
BOEVANGE/ATTERT	3 (65.66)	14.14 (0.80, 251.29)	1.04 (0.74, 1.66)
BOURSCHEID	2 (100.00)	86466749.73 (0.00, Infinity)	1.06 (0.78, 1.62)
BOUS	2 (100.00)	48614120.69 (0.00, Infinity)	1.03 (0.68, 1.66)
CLERVAUX	5 (34.33)	1.87 (0.48, 7.19)	1.02 (0.68, 1.60)
COLMAR-BERG	3 (23.83)	0.94 (0.19, 4.74)	0.98 (0.66, 1.43)
CONSDORF	1 (24.55)	0.35 (0.03, 3.70)	0.97 (0.61, 1.43)
CONTERN	5 (33.56)	1.44 (0.33, 6.28)	0.97 (0.65, 1.43)
DALHEIM	1 (12.95)	0.44 (0.05, 4.10)	0.95 (0.61, 1.37)
DIEKIRCH	7 (35.19)	2.12 (0.69, 6.56)	1.06 (0.76, 1.63)
DIFFERDANGE	14 (29.79)	1.46 (0.62, 3.40)	1.01 (0.71, 1.48)
DIPPACH	4 (21.57)	0.80 (0.21, 3.02)	0.96 (0.64, 1.34)
DUDELANGE	29 (44.37)	1.76 (0.90, 3.44)	1.08 (0.80, 1.58)
ECHTERNACH	8 (49.99)	2.99 (0.78, 11.48)	1.07 (0.73, 1.78)
ELL	1 (100.00)	35042147.99 (0.00, Infinity)	1.02 (0.68, 1.64)
ERPELDANGE	2 (46.84)	5.49 (0.60, 50.14)	1.03 (0.69, 1.60)
ESCH/ALZETTE	33 (37.48)	1.79 (0.95, 3.37)	1.10 (0.79, 1.62)
ESCH/SURE	2 (33.51)	1.52 (0.24, 9.74)	1.00 (0.67, 1.53)
ETTELBRUCK	7 (24.33)	0.65 (0.22, 1.98)	0.93 (0.60, 1.28)
FEULEN	0 (0.00)	0.00 (0.00, Infinity)	0.95 (0.60, 1.34)
FISCHBACH	1 (20.22)	0.96 (0.07, 12.71)	1.00 (0.67, 1.48)
FLAXWEILER	2 (38.62)	2.15 (0.25, 18.11)	0.99 (0.62, 1.50)
FRISANGE	4 (20.34)	0.68 (0.19, 2.52)	0.92 (0.55, 1.28)
GARNICH	3 (60.46)	3.25 (0.42, 25.43)	1.05 (0.72, 1.70)
GOESDORF	4 (47.74)	2.80 (0.64, 12.26)	1.05 (0.71, 1.60)
GREVENMACHER	2 (8.59)	0.20 (0.04, 1.02)	0.87 (0.50, 1.21)
GROSBOUS	2 (70.84)	18.06 (1.20, 272.62)	1.03 (0.70, 1.58)
HEFFINGEN	0 (0.00)	0.00 (0.00, Infinity)	0.99 (0.65, 1.44)
HESPERANGE	14 (33.65)	0.99 (0.43, 2.31)	0.93 (0.61, 1.27)
HOBSCHEID	4 (36.24)	0.92 (0.23, 3.72)	0.97 (0.63, 1.39)
JUNGLINSTER	7 (37.43)	1.87 (0.57, 6.09)	1.01 (0.71, 1.43)
KAERJEN	10 (31.18)	0.88 (0.34, 2.24)	0.95 (0.63, 1.33)
KAYL	15 (39.94)	2.03 (0.85, 4.85)	1.10 (0.77, 1.70)
KEHLEN	8 (53.45)	2.65 (0.79, 8.95)	1.05 (0.76, 1.59)

KIISCHPELT	0 (0.00)	0.00 (0.00, Infinity)	1.00 (0.67, 1.47)
KOERICH	8 (64.66)	4.59 (1.29, 16.35)	1.10 (0.78, 1.79)
KOPSTAL	4 (35.40)	0.57 (0.14, 2.29)	0.91 (0.59, 1.27)
LAC DE LA HAUTE SURE	2 (28.66)	0.84 (0.11, 6.56)	0.99 (0.66, 1.49)
LAROCHETTE	6 (42.37)	2.58 (0.63, 10.58)	1.05 (0.73, 1.56)
LENNINGEN	4 (44.07)	2.27 (0.55, 9.33)	1.01 (0.70, 1.52)
LEUDELANGE	1 (26.49)	1.64 (0.14, 19.46)	1.00 (0.70, 1.54)
LINTGEN	7 (70.53)	2.86 (0.65, 12.55)	1.06 (0.74, 1.72)
LORENTZWEILER	4 (39.27)	1.12 (0.23, 5.43)	0.99 (0.68, 1.44)
MAMER	12 (47.89)	4.00 (1.29, 12.43)	1.11 (0.80, 1.82)
MANTERNACH	0 (0.00)	0.00 (0.00, Infinity)	0.98 (0.62, 1.46)
MERSCH	11 (39.29)	1.26 (0.48, 3.30)	0.99 (0.70, 1.39)
MERTERT	9 (57.23)	6.95 (1.72, 28.05)	1.12 (0.76, 1.90)
MERTZIG	1 (18.72)	0.92 (0.08, 10.70)	0.98 (0.64, 1.43)
MOMPACH	2 (76.48)	5.60 (0.37, 84.81)	1.05 (0.69, 1.75)
MONDERCANGE	8 (38.03)	0.92 (0.31, 2.76)	0.98 (0.67, 1.40)
MONDORF-LES-BAINS	6 (48.93)	2.61 (0.65, 10.54)	1.03 (0.66, 1.74)
NIEDERANVEN	6 (27.35)	0.49 (0.16, 1.57)	0.87 (0.54, 1.22)
NOMMERN	2 (51.93)	4.89 (0.18, 130.19)	1.02 (0.69, 1.54)
PARC HOSINGEN	5 (39.27)	1.70 (0.35, 8.16)	1.02 (0.69, 1.63)
PETANGE	13 (29.37)	1.70 (0.71, 4.06)	1.05 (0.73, 1.56)
PREIZERDAUL	0 (0.00)	0.00 (0.00, Infinity)	0.97 (0.60, 1.45)
PUTSCHEID	1 (40.41)	1.70 (0.08, 36.11)	1.02 (0.64, 1.61)
RAMBROUCH	3 (57.10)	1.43 (0.24, 8.45)	0.99 (0.67, 1.51)
RECKANGE/MESS	2 (13.19)	0.49 (0.09, 2.79)	0.96 (0.61, 1.39)
REDANGE	3 (34.46)	1.37 (0.27, 7.03)	0.99 (0.65, 1.54)
REISDORF	0 (0.00)	0.00 (0.00, Infinity)	1.00 (0.62, 1.52)
REMICH	4 (68.64)	4.53 (0.66, 31.07)	1.06 (0.70, 1.82)
ROESER	10 (43.84)	1.93 (0.63, 5.92)	1.01 (0.72, 1.44)
ROSPORT	4 (36.58)	4.00 (0.80, 20.09)	1.07 (0.74, 1.77)
RUMELANGE	3 (26.85)	1.56 (0.29, 8.54)	1.05 (0.67, 1.65)
SAEUL	0 (0.00)	0.00 (0.00, Infinity)	1.00(0.67, 1.46)
SANDWEILER	4 (28.19)	0.92 (0.21, 4.10)	0.94 (0.60, 1.33)
SANEM	18 (36.87)	1.47 (0.67, 3.20)	1.02 (0.73, 1.43)
SCHENGEN	3 (19.75)	0.98 (0.23, 4.24)	0.98 (0.64, 1.49)
SCHIEREN	3 (58.23)	13.46 (0.50, 365.19)	1.05 (0.73, 1.73)
SCHIFFLANGE	11 (58.48)	4.11 (1.32, 12.78)	1.16 (0.80, 1.88)
SCHUTTRANGE	2 (16.04)	0.84 (0.14, 4.95)	0.94 (0.59, 1.36)
STADTBREDIMUS	0 (0.00)	0.00 (0.00, Infinity)	0.99 (0.64, 1.52)
STEINFORT	1 (12.22)	0.58 (0.07, 5.13)	0.97 (0.60, 1.45)
STEINSEL	5 (36.77)	0.87 (0.24, 3.22)	0.94 (0.63, 1.31)
STRASSEN	4 (27.05)	0.71 (0.17, 2.96)	0.94 (0.59, 1.34)
TANDEL	5 (61.23)	6.19 (1.04, 36.88)	1.09 (0.78, 1.81)
TROISVIERGES	3 (26.75)	0.97 (0.19, 4.90)	0.96 (0.54, 1.51)
TUNTANGE	0 (0.00)	0.00 (0.00, Infinity)	0.98 (0.68, 1.52)
USELDANGE	2 (39.06)	1.68 (0.22, 12.97)	1.01 (0.68, 1.53)
VALLéE DE L'ERNZ	3 (15.00)	1.32 (0.30, 5.79)	1.00 (0.67, 1.42)
VIANDEN	2 (100.00)	16399186.82 (0.00, Infinity)	1.08 (0.68, 1.91)
WAHL	1 (46.07)	0.61 (0.04, 9.08)	0.98 (0.62, 1.45)
WALDBILLIG	1 (48.08)	1.87 (0.06, 59.48)	1.00 (0.64, 1.51)
WALDBREDIMUS	2 (100.00)	5714113.56 (0.00, Infinity)	1.01 (0.67, 1.66)
WALFERDANGE	11 (33.20)	1.10 (0.43, 2.82)	0.93 (0.61, 1.30)
WEILER-LA-TOUR	1 (25.03)	0.90 (0.08, 9.85)	0.96 (0.61, 1.45)

WEISWAMPACH	0 (0.00)	0.00 (0.00, Infinity)	0.98 (0.56, 1.55)
WILTZ	8 (36.63)	1.36 (0.45, 4.13)	1.00 (0.68, 1.53)
WINCRANGE	0 (0.00)	0.00 (0.00, Infinity)	0.93 (0.54,1.33)
WINSELER	3 (65.24)	8.31 (0.61, 112.47)	1.03 (0.66, 1.68)
WORMELDANGE	1 (26.82)	0.69 (0.07, 6.95)	0.96 (0.60, 1.48)

† $p = 0.04$.

Table S9. High plasma fasting glucose (diabetes) according to cantons.

	**Crude prevalence N (%)**	**Age- and sex-adjusted OR (95% CI)**	**Age- and sex-adjusted POR (95% CI)**
Age, mean (SE), years	57.37 (1.35)	1.11 (1.08, 1.13)	1.11 (1.08, 1.14)
Sex			
Male	39 (5.18)	1.00	1.00
Female	30 (3.54)	0.68 (0.41, 1.14)	0.69 (0.41, 1.16)
Canton†			
LUXEMBOURG	23 (5.45)	1.00	1.11 (0.83, 1.82)
CLERVAUX	2 (2.71)	0.82 (0.17, 3.89)	0.99 (0.58, 1.75)
DIEKIRCH	3 (3.00)	0.60 (0.17, 2.13)	0.97 (0.63, 1.39)
REDANGE	3 (5.69)	1.50 (0.40, 5.57)	1.06 (0.71, 1.83)
VIANDEN	0 (0.00)	0.00 (0.00, Infinity)	0.95 (0.53, 1.64)
WILTZ	2 (4.55)	0.73 (0.16, 3.43)	0.98 (0.60, 1.56)
ECHTERNACH	1 (2.49)	0.37 (0.05, 2.94)	0.96 (0.58, 1.37)
GREVENMACHER	3 (3.37)	0.62 (0.17, 2.23)	0.99 (0.63, 1.48)
REMICH	1 (1.35)	0.34 (0.04, 2.69)	0.96 (0.53, 1.53)
CAPELLEN	5 (3.78)	0.57 (0.20, 1.57)	0.96 (0.60, 1.41)
ESCH/ALZETTE	19 (4.02)	0.74 (0.39, 1.42)	0.99 (0.70, 1.42)
MERSCH	7 (7.47)	1.28 (0.51, 3.25)	1.09 (0.79, 1.84)

† $p = 0.77$

Table S10. High plasma fasting glucose (diabetes) according to municipalities.

	**Crude prevalence N (%)**	**Age- and sex-adjusted OR (95% CI)**	**Age- and sex-adjusted POR (95% CI)**
Municipality †			
LUXEMBOURG	8 (3.96)	1.00	0.98 (0.67, 1.42)
BEAUFORT	0 (0.00)	0.00 (0.00, Infinity)	0.99 (0.58, 1.69)
BECH	0 (0.00)	0.00 (0.00, Infinity)	1.00 (0.63, 1.68)
BECKERICH	1 (9.63)	5.43 (0.54, 54.19)	1.06 (0.69, 2.03)
BERDORF	0 (0.00)	0.00 (0.00, Infinity)	0.98 (0.57, 1.67)
BERTRANGE	0 (0.00)	0.00 (0.00, Infinity)	0.94 (0.54, 1.38)
BETTEMBOURG	1 (3.04)	0.86 (0.10, 7.65)	0.98 (0.57, 1.57)
BETTENDORF	1 (6.93)	1.33 (0.14, 13.12)	1.00 (0.63, 1.66)
BETZDORF	0 (0.00)	0.00 (0.00, Infinity)	0.98 (0.57, 1.57)
BISSEN	0 (0.00)	0.00 (0.00, Infinity)	1.02 (0.61, 1.78)
BIWER	0 (0.00)	0.00 (0.00, Infinity)	0.98 (0.56, 1.56)
BOEVANGE/ATTERT	1 (22.82)	12.98 (1.04, 161.18)	1.06 (0.67, 1.91)
BOURSCHEID	0 (0.00)	0.00 (0.00, Infinity)	0.99 (0.60, 1.62)
BOUS	0 (0.00)	0.00 (0.00, Infinity)	0.98 (0.53, 1.73)
CLERVAUX	1 (4.08)	2.33 (0.24, 23.08)	1.00 (0.58, 1.77)
COLMAR-BERG	1 (9.04)	3.37 (0.33, 34.24)	1.02 (0.65, 1.76)
CONSDORF	0 (0.00)	0.00 (0.00, Infinity)	1.00 (0.60, 1.64)
CONTERN	1 (7.54)	1.39 (0.13, 14.49)	1.01 (0.63, 1.67)
DALHEIM	0 (0.00)	0.00 (0.00, Infinity)	0.98 (0.59, 1.60)

DIEKIRCH	1 (6.40)	2.49 (0.27, 22.68)	1.01 (0.65, 1.83)
DIFFERDANGE	3 (6.22)	2.14 (0.49, 9.40)	1.05 (0.62, 1.88)
DIPPACH	0 (0.00)	0.00 (0.00, Infinity)	0.97 (0.57, 1.63)
DUDELANGE	2 (3.32)	0.64 (0.13, 3.25)	0.94 (0.58, 1.41)
ECHTERNACH	0 (0.00)	0.00 (0.00, Infinity)	0.96 (0.54, 1.59)
ELL	0 (0.00)	0.00 (0.00, Infinity)	1.02 (0.59, 1.94)
ERPELDANGE	0 (0.00)	0.00 (0.00, Infinity)	0.99 (0.59, 1.63)
ESCH/ALZETTE	3 (3.30)	0.89 (0.22, 3.64)	0.97 (0.60, 1.57)
ESCH/SURE	0 (0.00)	0.00 (0.00, Infinity)	1.00 (0.64, 1.64)
ETTELBRUCK	0 (0.00)	0.00 (0.00, Infinity)	0.93 (0.52, 1.39)
FEULEN	0 (0.00)	0.00 (0.00, Infinity)	0.99 (0.57, 1.67)
FISCHBACH	0 (0.00)	0.00 (0.00, Infinity)	1.00 (0.63, 1.71)
FLAXWEILER	0 (0.00)	0.00 (0.00, Infinity)	1.00 (0.56, 1.63)
FRISANGE	1 (5.33)	2.29 (0.24, 21.60)	1.02 (0.63, 1.80)
GARNICH	1 (24.22)	4.78 (0.34, 67.15)	1.04 (0.65, 1.77)
GOESDORF	1 (16.27)	6.09 (0.61, 60.42)	1.04 (0.62, 1.81)
GREVENMACHER	2 (8.59)	3.38 (0.59, 19.53)	1.06 (0.69, 1.89)
GROSBOUS	0 (0.00)	0.00 (0.00, Infinity)	1.00 (0.56, 1.66)
HEFFINGEN	0 (0.00)	0.00 (0.00, Infinity)	1.00 (0.64, 1.63)
HESPERANGE	4 (9.06)	2.34 (0.61, 8.90)	1.09 (0.74, 1.93)
HOBSCHEID	2 (18.51)	5.49 (0.89, 33.83)	1.08 (0.71, 2.15)
JUNGLINSTER	0 (0.00)	0.00 (0.00, Infinity)	0.97 (0.52, 1.49)
KAERJEN	1 (3.85)	0.81 (0.09, 7.11)	0.98 (0.58, 1.62)
KAYL	0 (0.00)	0.00 (0.00, Infinity)	0.93 (0.52, 1.46)
KEHLEN	0 (0.00)	0.00 (0.00, Infinity)	0.98 (0.61, 1.44)
KIISCHPELT	0 (0.00)	0.00 (0.00, Infinity)	0.99 (0.58, 1.57)
KOERICH	0 (0.00)	0.00 (0.00, Infinity)	0.99 (0.60, 1.56)
KOPSTAL	0 (0.00)	0.00 (0.00, Infinity)	0.96 (0.57, 1.46)
LAC DE LA HAUTE SURE	1 (10.43)	4.67 (0.39, 56.08)	1.02 (0.59, 1.84)
LAROCHETTE	0 (0.00)	0.00 (0.00, Infinity)	0.96 (0.52, 1.51)
LENNINGEN	0 (0.00)	0.00 (0.00, Infinity)	0.99 (0.59, 1.67)
LEUDELANGE	0 (0.00)	0.00 (0.00, Infinity)	0.98 (0.58, 1.58)
LINTGEN	2 (21.57)	4.90 (0.78, 30.90)	1.09 (0.71, 2.27)
LORENTZWEILER	2 (21.47)	4.33 (0.67, 28.03)	1.08 (0.75, 1.95)
MAMER	1 (3.79)	0.70 (0.07, 6.51)	0.98 (0.59, 1.59)
MANTERNACH	0 (0.00)	0.00 (0.00, Infinity)	0.99 (0.54, 1.68)
MERSCH	1 (3.25)	0.63 (0.07, 5.61)	1.00 (0.63, 1.48)
MERTERT	1 (4.63)	1.47 (0.14, 15.22)	1.01 (0.59, 1.75)
MERTZIG	0 (0.00)	0.00 (0.00, Infinity)	1.00 (0.60, 1.61)
MOMPACH	0 (0.00)	0.00 (0.00, Infinity)	0.97 (0.52, 1.62)
MONDERCANGE	3 (13.56)	2.71 (0.60, 12.25)	1.05 (0.70, 1.87)
MONDORF-LES-BAINS	0 (0.00)	0.00 (0.00, Infinity)	0.96 (0.49, 1.52)
NIEDERANVEN	2 (7.30)	1.50 (0.27, 8.49)	1.02 (0.69, 1.66)
NOMMERN	0 (0.00)	0.00 (0.00, Infinity)	1.00 (0.62, 1.63)
PARC HOSINGEN	1 (6.69)	1.77 (0.15, 20.39)	1.01 (0.61, 1.71)
PETANGE	1 (2.21)	0.82 (0.09, 7.19)	0.98 (0.52, 1.67)
PREIZERDAUL	0 (0.00)	0.00 (0.00, Infinity)	1.01 (0.60, 1.74)
PUTSCHEID	0 (0.00)	0.00 (0.00, Infinity)	1.01 (0.56, 1.70)
RAMBROUCH	1 (13.13)	3.20 (0.26, 38.91)	1.05 (0.67, 1.75)
RECKANGE/MESS	0 (0.00)	0.00 (0.00, Infinity)	0.96 (0.54, 1.49)
REDANGE	0 (0.00)	0.00 (0.00, Infinity)	1.01 (0.59, 1.76)
REISDORF	0 (0.00)	0.00 (0.00, Infinity)	0.99 (0.56, 1.63)
REMICH	0 (0.00)	0.00 (0.00, Infinity)	0.96 (0.51, 1.73)

ROESER	1 (4.46)	0.84 (0.09, 7.48)	0.99 (0.62, 1.60)
ROSPORT	0 (0.00)	0.00 (0.00, Infinity)	0.98 (0.53, 1.73)
RUMELANGE	1 (11.05)	5.98 (0.51, 70.59)	1.02 (0.61, 1.96)
SAEUL	0 (0.00)	0.00 (0.00, Infinity)	1.02 (0.62, 1.70)
SANDWEILER	2 (13.52)	4.39 (0.64, 30.13)	1.07 (0.71, 2.05)
SANEM	1 (2.11)	0.49 (0.06, 4.17)	0.94 (0.53, 1.42)
SCHENGEN	1 (5.65)	3.47 (0.35, 34.09)	1.02 (0.59, 1.88)
SCHIEREN	0 (0.00)	0.00 (0.00, Infinity)	0.98 (0.56, 1.52)
SCHIFFLANGE	2 (10.11)	2.40 (0.41, 14.02)	1.04 (0.64, 1.98)
SCHUTTRANGE	0 (0.00)	0.00 (0.00, Infinity)	0.98 (0.57, 1.58)
STADTBREDIMUS	0 (0.00)	0.00 (0.00, Infinity)	0.99 (0.49, 1.67)
STEINFORT	0 (0.00)	0.00 (0.00, Infinity)	0.99 (0.56, 1.55)
STEINSEL	2 (15.87)	3.40 (0.56, 20.50)	1.05 (0.70, 1.95)
STRASSEN	1 (7.29)	1.88 (0.19, 18.85)	1.03 (0.68, 1.78)
TANDEL	0 (0.00)	0.00 (0.00, Infinity)	0.98 (0.57, 1.63)
TROISVIERGES	0 (0.00)	0.00 (0.00, Infinity)	0.95 (0.45, 1.72)
TUNTANGE	0 (0.00)	0.00 (0.00, Infinity)	1.01 (0.63, 1.68)
USELDANGE	1 (21.13)	8.88 (0.74, 106.58)	1.05 (0.68, 1.93)
VALLéE DE L'ERNZ	1 (6.49)	5.33 (0.53, 53.74)	1.03 (0.71, 1.74)
VIANDEN	0 (0.00)	0.00 (0.00, Infinity)	0.98 (0.50, 1.74)
WAHL	0 (0.00)	0.00 (0.00, Infinity)	1.00 (0.58, 1.61)
WALDBILLIG	1 (48.08)	32.87 (0.56, 1918.40)	1.03 (0.63, 1.80)
WALDBREDIMUS	0 (0.00)	0.00 (0.00, Infinity)	0.98 (0.57, 1.65)
WALFERDANGE	3 (8.33)	2.29 (0.51, 10.25)	1.08 (0.72, 2.06)
WEILER-LA-TOUR	0 (0.00)	0.00 (0.00, Infinity)	0.99 (0.58, 1.64)
WEISWAMPACH	0 (0.00)	0.00 (0.00, Infinity)	0.98 (0.49, 1.94)
WILTZ	0 (0.00)	0.00 (0.00, Infinity)	0.96 (0.51, 1.51)
WINCRANGE	0 (0.00)	0.00 (0.00, Infinity)	0.97 (0.52, 1.64)
WINSELER	0 (0.00)	0.00 (0.00, Infinity)	0.98 (0.51, 1.64)
WORMELDANGE	0 (0.00)	0.00 (0.00, Infinity)	1.00 (0.57, 1.64)

† $p = 0.91$.

Table S11. Alcohol use according to cantons.

	**Crude prevalence N (%)**	**Age- and sex-adjusted OR (95% CI)**	**Age- and sex-adjusted POR (95% CI)**
Age, mean (SE), years	42.45 (0.2)	1.01 (1.00, 1.02)	1.01 (1.00, 1.02)
Sex			
Male	588 (89.09)	1.00	1.00
Female	533 (75.75)	0.38 (0.28, 0.52)	0.38 (0.28, 0.51)
Canton†			
LUXEMBOURG	322 (86.22)	1.00	1.19 (0.91, 1.67)
CLERVAUX	32 (83.71)	0.63 (0.27, 1.48)	0.91 (0.57, 1.42)
DIEKIRCH	65 (82.53)	0.76 (0.39, 1.49)	0.98 (0.69, 1.41)
REDANGE	24 (71.04)	0.47 (0.20, 1.09)	0.87 (0.48, 1.26)
VIANDEN	9 (80.06)	0.62 (0.13, 3.03)	0.94 (0.49, 1.72)
WILTZ	31 (75.23)	0.41 (0.19, 0.91)	0.81 (0.47, 1.22)
ECHTERNACH	37 (84.62)	1.08 (0.40, 2.92)	1.13 (0.74, 1.99)
GREVENMACHER	63 (90.65)	1.71 (0.70, 4.19)	1.28 (0.90, 2.31)
REMICH	42 (84.57)	0.90 (0.38, 2.14)	1.08 (0.71, 1.73)
CAPELLEN	106 (84.37)	0.89 (0.50, 1.59)	1.06 (0.78, 1.58)
ESCH/ALZETTE	318 (77.90)	0.58 (0.39, 0.85)	0.82 (0.59, 1.05)
MERSCH	72 (83.37)	0.75 (0.39, 1.45)	1.02 (0.71, 1.52)

† $p = 0.09$.

Table S12. Alcohol use according to municipalities.

	Crude prevalence N (%)	Age- and sex-adjusted OR (95% CI)	Age- and sex-adjusted POR (95% CI)
Municipality †			
LUXEMBOURG	166 (86.04)	1.00	1.14 (0.89, 1.57)
BEAUFORT	4 (100.00)	5298621.59 (0.00, Infinity)	1.04 (0.64, 1.70)
BECH	1 (100.00)	3168647.28 (0.00, Infinity)	1.08 (0.72, 1.81)
BECKERICH	5 (61.98)	0.30 (0.07, 1.40)	0.91 (0.52, 1.35)
BERDORF	6 (100.00)	4193025.02 (0.00, Infinity)	1.06 (0.68, 1.79)
BERTRANGE	27 (88.22)	1.36 (0.38, 4.88)	1.08 (0.74, 1.64)
BETTEMBOURG	20 (78.27)	0.55 (0.20, 1.53)	0.92 (0.57, 1.36)
BETTENDORF	9 (92.22)	1.15 (0.13, 9.75)	1.01 (0.66, 1.68)
BETZDORF	5 (100.00)	8032008.23 (0.00, Infinity)	1.14 (0.76, 1.95)
BISSEN	3 (69.46)	0.19 (0.03, 1.29)	0.94 (0.55, 1.39)
BIWER	6 (100.00)	4931945.60 (0.00, Infinity)	1.14 (0.76, 2.02)
BOEVANGE/ATTERT	3 (65.66)	0.45 (0.04, 4.79)	0.96 (0.59, 1.47)
BOURSCHEID	2 (100.00)	6445021.27 (0.00, Infinity)	0.97 (0.63, 1.50)
BOUS	2 (100.00)	6247790.49 (0.00, Infinity)	1.09 (0.66, 2.03)
CLERVAUX	12 (91.22)	1.13 (0.23, 5.41)	0.97 (0.58, 1.50)
COLMAR-BERG	8 (73.73)	0.47 (0.09, 2.44)	0.96 (0.59, 1.44)
CONSDORF	4 (100.00)	7319898.43 (0.00, Infinity)	1.07 (0.68, 1.76)
CONTERN	10 (82.19)	0.81 (0.16, 4.01)	1.09 (0.72, 1.70)
DALHEIM	7 (80.27)	1.22 (0.14, 10.54)	1.08 (0.71, 1.81)
DIEKIRCH	13 (94.92)	2.12 (0.26, 17.14)	1.04 (0.68, 1.68)
DIFFERDANGE	27 (73.01)	0.38 (0.16, 0.89)	0.80 (0.44, 1.26)
DIPPACH	12 (85.87)	0.90 (0.19, 4.33)	0.99 (0.65, 1.57)
DUDELANGE	46 (81.41)	0.73 (0.32, 1.64)	0.94 (0.65, 1.36)
ECHTERNACH	11 (71.78)	0.46 (0.11, 1.82)	1.03 (0.62, 1.69)
ELL	0 (0.00)	0.00 (0.00, Infinity)	0.90 (0.49, 1.45)
ERPELDANGE	4 (100.00)	6486618.64 (0.00, Infinity)	1.00 (0.62, 1.58)
ESCH/ALZETTE	56 (74.17)	0.42 (0.21, 0.82)	0.79 (0.49, 1.12)
ESCH/SURE	5 (86.95)	0.78 (0.08, 7.26)	0.94 (0.58, 1.43)
ETTELBRUCK	18 (73.61)	0.45 (0.16, 1.29)	0.91 (0.57, 1.37)
FEULEN	2 (100.00)	5599282.27 (0.00, Infinity)	0.97 (0.58, 1.58)
FISCHBACH	3 (100.00)	7648292.85 (0.00, Infinity)	1.07 (0.71, 1.72)
FLAXWEILER	4 (100.00)	7601544.47 (0.00, Infinity)	1.14 (0.73, 2.00)
FRISANGE	15 (86.69)	1.13 (0.24, 5.36)	1.06 (0.66, 1.84)
GARNICH	4 (81.77)	0.63 (0.06, 6.16)	0.97 (0.59, 1.57)
GOESDORF	7 (83.73)	1.03 (0.12, 8.97)	0.93 (0.58, 1.49)
GREVENMACHER	16 (86.91)	1.39 (0.30, 6.51)	1.15 (0.77, 1.97)
GROSBOUS	3 (100.00)	10139739.84 (0.00, Infinity)	1.00 (0.63, 1.68)
HEFFINGEN	1 (100.00)	3542440.15 (0.00, Infinity)	1.04 (0.68, 1.70)
HESPERANGE	35 (93.95)	2.66 (0.60, 11.87)	1.19 (0.84, 1.97)
HOBSCHEID	11 (100.00)	5775714.27 (0.00, Infinity)	1.05 (0.70, 1.72)
JUNGLINSTER	15 (95.25)	2.12 (0.26, 17.00)	1.14 (0.80, 1.83)
KAERJEN	23 (80.23)	0.67 (0.23, 1.96)	0.93 (0.61, 1.34)
KAYL	23 (73.48)	0.46 (0.17, 1.20)	0.86 (0.52, 1.31)
KEHLEN	13 (83.68)	0.99 (0.21, 4.77)	1.06 (0.74, 1.63)
KIISCHPELT	1 (100.00)	2858712.98 (0.00, Infinity)	0.92 (0.53, 1.52)
KOERICH	9 (83.84)	0.69 (0.14, 3.49)	1.01 (0.63, 1.62)
KOPSTAL	9 (90.39)	1.47 (0.18, 12.39)	1.09 (0.72, 1.78)
LAC DE LA HAUTE SURE	4 (69.30)	0.28 (0.05, 1.72)	0.89 (0.51, 1.42)

LAROCHETTE	9 (80.91)	0.33 (0.08, 1.34)	0.94 (0.55, 1.40)
LENNINGEN	8 (100.00)	4775151.81 (0.00, Infinity)	1.13 (0.71, 1.94)
LEUDELANGE	3 (100.00)	5231904.43 (0.00, Infinity)	1.02 (0.67, 1.55)
LINTGEN	8 (79.15)	0.53 (0.10, 2.76)	1.01 (0.62, 1.58)
LORENTZWEILER	8 (84.20)	1.49 (0.18, 12.63)	1.08 (0.73, 1.67)
MAMER	18 (83.04)	0.75 (0.23, 2.44)	1.01 (0.67, 1.53)
MANTERNACH	3 (100.00)	3535296.02 (0.00, Infinity)	1.12 (0.71, 2.03)
MERSCH	24 (89.11)	1.26 (0.35, 4.55)	1.05 (0.75, 1.52)
MERTERT	11 (85.49)	0.92 (0.19, 4.49)	1.11 (0.70, 2.06)
MERTZIG	4 (100.00)	4823831.00 (0.00, Infinity)	0.98 (0.62, 1.55)
MOMPACH	2 (62.02)	0.33 (0.03, 4.06)	1.06 (0.63, 1.90)
MONDERCANGE	17 (86.68)	0.89 (0.24, 3.32)	0.96 (0.62, 1.50)
MONDORF-LES-BAINS	9 (75.60)	0.43 (0.11, 1.77)	1.00 (0.56, 1.82)
NIEDERANVEN	19 (92.83)	1.29 (0.28, 5.98)	1.12 (0.78, 1.77)
NOMMERN	3 (100.00)	3327759.84 (0.00, Infinity)	1.00 (0.63, 1.54)
PARC HOSINGEN	6 (58.32)	0.13 (0.03, 0.50)	0.84 (0.44, 1.29)
PETANGE	29 (79.13)	0.51 (0.21, 1.27)	0.85 (0.50, 1.33)
PREIZERDAUL	1 (100.00)	3603736.92 (0.00, Infinity)	0.96 (0.58, 1.50)
PUTSCHEID	2 (100.00)	5780138.87 (0.00, Infinity)	0.96 (0.54, 1.60)
RAMBROUCH	4 (79.02)	0.60 (0.06, 5.88)	0.93 (0.56, 1.53)
RECKANGE/MESS	11 (89.08)	1.73 (0.21, 14.31)	1.01 (0.65, 1.62)
REDANGE	5 (56.39)	0.30 (0.07, 1.38)	0.89 (0.50, 1.38)
REISDORF	2 (100.00)	6645108.64 (0.00, Infinity)	1.01 (0.62, 1.69)
REMICH	4 (78.35)	0.52 (0.05, 5.18)	1.06 (0.62, 2.00)
ROESER	16 (76.52)	0.55 (0.18, 1.66)	1.00 (0.65, 1.47)
ROSPORT	8 (100.00)	7606824.56 (0.00, Infinity)	1.15 (0.72, 2.27)
RUMELANGE	6 (61.63)	0.42 (0.10, 1.84)	0.86 (0.49, 1.42)
SAEUL	1 (100.00)	10868813.62 (0.00, Infinity)	0.99 (0.64, 1.56)
SANDWEILER	8 (69.00)	0.23 (0.06, 0.85)	0.99 (0.60, 1.51)
SANEM	35 (78.22)	0.70 (0.29, 1.71)	0.93 (0.63, 1.37)
SCHENGEN	9 (84.32)	0.77 (0.15, 3.86)	1.05 (0.62, 1.86)
SCHIEREN	4 (100.00)	6225051.77 (0.00, Infinity)	1.00 (0.65, 1.65)
SCHIFFLANGE	14 (78.16)	0.53 (0.16, 1.79)	0.88 (0.51, 1.42)
SCHUTTRANGE	9 (75.72)	0.80 (0.16, 4.05)	1.09 (0.73, 1.73)
STADTBREDIMUS	1 (100.00)	3960327.89 (0.00, Infinity)	1.09 (0.64, 1.91)
STEINFORT	7 (79.15)	0.66 (0.13, 3.48)	0.98 (0.60, 1.56)
STEINSEL	10 (78.49)	0.48 (0.12, 1.92)	1.05 (0.70, 1.63)

STRASSEN	9 (79.89)	0.51 (0.10, 2.61)	1.03 (0.63, 1.67)
TANDEL	6 (91.45)	0.81 (0.09, 7.27)	0.98 (0.61, 1.57)
TROISVIERGES	9 (87.33)	0.80 (0.16, 4.06)	0.97 (0.51, 1.83)
TUNTANGE	2 (100.00)	6358737.11 (0.00, Infinity)	1.03 (0.65, 1.68)
USELDANGE	2 (66.14)	0.47 (0.04, 5.37)	0.95 (0.58, 1.48)
VALLéE DE L'ERNZ	7 (57.69)	0.18 (0.05, 0.65)	0.90 (0.55, 1.35)
VIANDEN	1 (33.51)	0.12 (0.01, 2.16)	0.93 (0.45, 1.66)
WAHL	3 (100.00)	4931291.03 (0.00, Infinity)	0.98 (0.60, 1.58)
WALDBILLIG	1 (48.08)	0.13 (0.01, 2.25)	0.99 (0.60, 1.63)
WALDBREDIMUS	2 (100.00)	2860305.76 (0.00, Infinity)	1.11 (0.71, 1.93)
WALFERDANGE	26 (90.48)	1.23 (0.34, 4.45)	1.14 (0.78, 1.98)
WEILER-LA-TOUR	3 (74.97)	0.44 (0.04, 4.68)	1.04 (0.60, 1.73)
WEISWAMPACH	1 (100.00)	10235408.74 (0.00, Infinity)	0.96 (0.52, 1.74)
WILTZ	10 (62.75)	0.18 (0.06, 0.56)	0.80 (0.42, 1.21)
WINCRANGE	4 (100.00)	6091782.32 (0.00, Infinity)	0.98 (0.59, 1.65)
WINSELER	4 (100.00)	4684948.28 (0.00, Infinity)	0.94 (0.54, 1.56)
WORMELDANGE	3 (68.62)	0.57 (0.05, 5.89)	1.09 (0.68, 1.90)

† $p = 0.56$.

Table S13. High total cholesterol according to cantons.

	**Crude prevalence N (%)**	**Age- and sex-adjusted OR (95% CI)**	**Age- and sex-adjusted POR (95% CI)**
Age, mean (SE), years	45.53 (0.33)	1.05(1.04,1.06)	1.05(1.04,1.06)
Sex			
Male	438 (59.65)	1.00	1.00
Female	468 (60.80)	1.04 (0.83, 1.30)	1.04 (0.84, 1.31)
Canton†			
LUXEMBOURG	240 (58.16)	1.00	0.95 (0.78, 1.12)
CLERVAUX	26 (55.36)	1.25 (0.62, 2.52)	1.01 (0.79, 1.34)
DIEKIRCH	51 (53.41)	1.06 (0.64, 1.77)	0.99 (0.79, 1.21)
REDANGE	24 (58.68)	1.01 (0.49, 2.06)	0.99 (0.78, 1.22)
VIANDEN	5 (49.25)	0.41(0.12,1.43)	0.94(0.63,1.23)
WILTZ	31 (69.08)	1.50 (0.74, 3.02)	1.04 (0.83, 1.34)
ECHTERNACH	26 (56.90)	0.95 (0.49, 1.86)	0.98 (0.76, 1.24)
GREVENMACHER	50 (65.03)	1.38 (0.79, 2.40)	1.04 (0.84, 1.35)
REMICH	38 (71.70)	1.77 (0.92, 3.40)	1.08 (0.86, 1.50)
CAPELLEN	83 (60.15)	1.05 (0.69, 1.62)	0.98 (0.79, 1.19)
ESCH/ALZETTE	274 (60.92)	1.16 (0.87, 1.57)	1.02 (0.86, 1.22)
MERSCH	58 (62.94)	1.20 (0.72, 2.01)	1.01 (0.83, 1.31)

† $p = 0.83$.

Table S14. High total cholesterol according to municipalities.

	Crude prevalence N (%)	Age- and sex-adjusted OR (95% CI)	Age- and sex-adjusted POR (95% CI)
Municipality †			
LUXEMBOURG	118 (55.29)	1.00	0.94 (0.75, 1.10)
BEAUFORT	2 (50.32)	1.20 (0.16, 9.01)	0.99 (0.78, 1.24)
BECH	1 (100.00)	8906603.38 (0.00, Infinity)	1.01 (0.80, 1.24)
BECKERICH	5 (61.44)	1.09 (0.25, 4.82)	0.99 (0.79, 1.28)
BERDORF	3 (41.42)	0.72 (0.13, 4.08)	0.99 (0.74, 1.25)
BERTRANGE	22 (70.54)	1.71 (0.74, 4.00)	1.01 (0.82, 1.25)
BETTEMBOURG	19 (59.42)	1.35 (0.58, 3.15)	1.00 (0.79, 1.24)
BETTENDORF	6 (67.53)	0.71 (0.18, 2.71)	0.98 (0.76, 1.23)
BETZDORF	6 (100.00)	8346657.82 (0.00, Infinity)	1.02 (0.82, 1.32)
BISSEN	4 (75.29)	4.29 (0.44, 41.94)	1.01 (0.82, 1.26)
BIWER	4 (58.82)	1.14 (0.18, 7.30)	1.00 (0.77, 1.31)
BOEVANGE/ATTERT	1 (21.48)	0.23 (0.02, 2.32)	0.98 (0.77, 1.23)
BOURSCHEID	2 (100.00)	15699063.65 (0.00, Infinity)	1.00 (0.80, 1.29)
BOUS	2 (100.00)	11397363.37 (0.00, Infinity)	1.03 (0.80, 1.33)
CLERVAUX	9 (63.43)	1.42 (0.44, 4.63)	1.00 (0.78, 1.28)
COLMAR-BERG	6 (50.74)	1.29 (0.32, 5.16)	0.99 (0.79, 1.24)
CONSDORF	3 (59.80)	0.78 (0.12, 5.09)	0.98 (0.76, 1.22)
CONTERN	9 (66.48)	1.83 (0.50, 6.69)	1.02 (0.82, 1.30)
DALHEIM	8 (100.00)	13449274.47 (0.00, Infinity)	1.05 (0.84, 1.39)
DIEKIRCH	9 (45.49)	0.85 (0.31, 2.39)	0.97 (0.73, 1.22)
DIFFERDANGE	24 (52.33)	1.16 (0.57, 2.37)	0.99 (0.76, 1.27)
DIPPACH	10 (54.47)	1.64 (0.51, 5.30)	1.01 (0.82, 1.29)
DUDELANGE	40 (63.31)	1.12 (0.60, 2.07)	0.97 (0.76, 1.20)
ECHTERNACH	7 (52.36)	0.67 (0.21, 2.09)	0.98 (0.75, 1.20)
ELL	1 (100.00)	12740528.18 (0.00, Infinity)	1.00 (0.78, 1.31)
ERPELDANGE	1 (13.81)	0.29 (0.03, 3.16)	0.98 (0.74, 1.21)
ESCH/ALZETTE	46 (57.11)	1.06 (0.61, 1.84)	0.97 (0.76, 1.16)
ESCH/SURE	6 (90.71)	6.11 (0.70, 53.60)	1.03 (0.83, 1.30)
ETTELBRUCK	16 (56.38)	1.25 (0.51, 3.09)	0.99 (0.80, 1.22)
FEULEN	2 (100.00)	5410149.87 (0.00, Infinity)	1.00 (0.79, 1.28)
FISCHBACH	2 (58.30)	0.71 (0.09, 5.34)	1.00 (0.80, 1.22)
FLAXWEILER	4 (100.00)	8521610.21 (0.00, Infinity)	1.03 (0.83, 1.36)
FRISANGE	10 (51.01)	1.13 (0.40, 3.21)	1.01 (0.79, 1.29)
GARNICH	3 (61.70)	0.79 (0.12, 5.06)	0.99 (0.79, 1.28)
GOESDORF	5 (53.56)	1.17 (0.26, 5.18)	1.00 (0.79, 1.28)
GREVENMACHER	13 (64.91)	1.79 (0.62, 5.16)	1.02 (0.83, 1.33)
GROSBOUS	2 (64.58)	1.71 (0.14, 21.74)	1.00 (0.79, 1.27)
HEFFINGEN	1 (100.00)	17316176.03 (0.00, Infinity)	1.00 (0.81, 1.27)
HESPERANGE	26 (62.71)	1.37 (0.64, 2.91)	1.00 (0.82, 1.25)
HOBSCHEID	7 (63.08)	1.02 (0.28, 3.70)	0.99 (0.76, 1.24)
JUNGLINSTER	11 (61.45)	1.59 (0.51, 4.95)	1.01 (0.82, 1.26)
KAERJEN	17 (59.36)	0.95 (0.41, 2.20)	0.98 (0.78, 1.22)
KAYL	21 (58.38)	1.23 (0.57, 2.68)	0.99 (0.77, 1.25)
KEHLEN	11 (69.48)	1.82 (0.53, 6.18)	1.01 (0.84, 1.27)
KIISCHPELT	1 (100.00)	4821509.14 (0.00, Infinity)	1.00 (0.76, 1.29)
KOERICH	11 (81.66)	3.90 (0.82, 18.47)	1.02 (0.81, 1.31)
KOPSTAL	9 (80.44)	1.94 (0.39, 9.55)	1.01 (0.81, 1.28)
LAC DE LA HAUTE SURE	5 (77.08)	3.42 (0.37, 31.32)	1.02 (0.81, 1.33)
LAROCHETTE	8 (65.86)	1.59 (0.44, 5.81)	1.00 (0.79, 1.24)

LENNINGEN	6 (64.86)	1.66 (0.40, 6.98)	1.02 (0.81, 1.31)
LEUDELANGE	3 (100.00)	13486867.82 (0.00, Infinity)	1.01 (0.81, 1.27)
LINTGEN	7 (69.28)	1.03 (0.25, 4.27)	0.99 (0.79, 1.26)
LORENTZWEILER	6 (62.73)	0.99 (0.22, 4.45)	0.99 (0.76, 1.22)
MAMER	12 (54.40)	0.81 (0.32, 2.08)	0.97 (0.78, 1.20)
MANTERNACH	2 (65.29)	2.21 (0.19, 25.57)	1.00 (0.79, 1.32)
MERSCH	21 (74.84)	2.14 (0.83, 5.52)	1.02 (0.84, 1.29)
MERTERT	8 (57.60)	0.86 (0.27, 2.71)	0.99 (0.75, 1.25)
MERTZIG	2 (33.33)	0.87 (0.11, 6.75)	0.99 (0.79, 1.22)
MOMPACH	3 (100.00)	8298312.95 (0.00, Infinity)	1.01 (0.79, 1.37)
MONDERCANGE	12 (55.38)	0.80 (0.29, 2.17)	0.98 (0.77, 1.21)
MONDORF-LES-BAINS	6 (51.27)	0.64 (0.19, 2.21)	0.99 (0.74, 1.30)
NIEDERANVEN	14 (54.68)	1.10 (0.42, 2.93)	1.00 (0.80, 1.23)
NOMMERN	1 (25.97)	0.32 (0.02, 4.14)	0.99 (0.77, 1.23)
PARC HOSINGEN	7 (66.83)	1.68 (0.39, 7.19)	1.01 (0.78, 1.31)
PETANGE	25 (59.58)	1.76 (0.82, 3.75)	1.03 (0.81, 1.41)
PREIZERDAUL	1 (40.40)	0.53 (0.03, 11.03)	0.99 (0.78, 1.28)
PUTSCHEID	0 (0.00)	0.00 (0.00, Infinity)	0.97 (0.74, 1.21)
RAMBROUCH	4 (57.82)	0.97 (0.17, 5.61)	1.00 (0.79, 1.26)
RECKANGE/MESS	8 (58.40)	1.79 (0.50, 6.42)	1.01 (0.81, 1.29)
REDANGE	6 (62.36)	1.47 (0.34, 6.40)	1.00 (0.79, 1.25)
REISDORF	2 (54.37)	2.58 (0.21, 31.32)	1.00 (0.79, 1.29)
REMICH	5 (80.96)	2.95 (0.33, 26.28)	1.03 (0.80, 1.41)
ROESER	16 (80.08)	2.71 (0.83, 8.90)	1.02 (0.84, 1.27)
ROSPORT	6 (61.32)	1.91 (0.44, 8.29)	1.01 (0.78, 1.30)
RUMELANGE	5 (51.44)	0.91 (0.22, 3.80)	0.99 (0.75, 1.27)
SAEUL	1 (100.00)	18631488.34 (0.00, Infinity)	1.01 (0.82, 1.31)
SANDWEILER	8 (61.89)	1.50 (0.41, 5.51)	1.01 (0.80, 1.25)
SANEM	30 (67.93)	1.45 (0.70, 2.99)	1.01 (0.81, 1.27)
SCHENGEN	9 (72.09)	1.99 (0.56, 7.04)	1.03 (0.78, 1.37)
SCHIEREN	3 (58.23)	2.13 (0.18, 25.51)	1.00 (0.77, 1.30)
SCHIFFLANGE	15 (79.01)	2.74 (0.84, 8.93)	1.02 (0.81, 1.36)
SCHUTTRANGE	7 (57.19)	1.64 (0.45, 6.01)	1.01 (0.81, 1.32)
STADTBREDIMUS	0 (0.00)	0.00 (0.00, Infinity)	1.01 (0.79, 1.32)
STEINFORT	3 (26.63)	0.46 (0.11, 1.93)	0.96 (0.74, 1.19)
STEINSEL	8 (63.96)	0.83 (0.25, 2.76)	0.99 (0.79, 1.20)
STRASSEN	7 (55.15)	0.94 (0.28, 3.19)	0.99 (0.79, 1.22)
TANDEL	3 (45.62)	0.45 (0.10, 2.16)	0.98 (0.74, 1.21)
TROISVIERGES	6 (37.53)	1.07 (0.29, 4.00)	1.00 (0.76, 1.30)
TUNTANGE	1 (31.98)	0.96 (0.05, 19.56)	1.00 (0.80, 1.26)
USELDANGE	2 (36.74)	0.42 (0.07, 2.69)	0.98 (0.78, 1.24)
VALLéE DE L'ERNZ	8 (50.15)	2.04 (0.57, 7.28)	1.01 (0.83, 1.29)
VIANDEN	2 (100.00)	6573668.96 (0.00, Infinity)	1.00 (0.74, 1.29)
WAHL	2 (53.93)	1.18 (0.09, 15.15)	1.00 (0.78, 1.29)
WALDBILLIG	1 (48.08)	0.56 (0.02, 12.82)	0.99 (0.77, 1.24)
WALDBREDIMUS	2 (100.00)	4876603.41 (0.00, Infinity)	1.02 (0.80, 1.32)
WALFERDANGE	20 (59.92)	1.28 (0.57, 2.91)	0.99 (0.77, 1.25)
WEILER-LA-TOUR	1 (24.29)	0.24 (0.02, 2.36)	0.99 (0.78, 1.24)
WEISWAMPACH	1 (100.00)	13024834.99 (0.00, Infinity)	1.00 (0.77, 1.32)
WILTZ	12 (65.32)	1.31 (0.45, 3.79)	1.00 (0.76, 1.31)
WINCRANGE	3 (51.41)	1.27 (0.18, 8.99)	1.00 (0.76, 1.29)
WINSELER	2 (47.53)	0.60 (0.07, 4.80)	0.99 (0.76, 1.26)
WORMELDANGE	2 (41.67)	0.44 (0.07, 2.93)	1.00 (0.78, 1.28)

† $p = 0.76$.

Table S15. Low glomerular filtration rate (chronic kidney disease) according to cantons.

	Crude prevalence N (%)	Age- and sex-adjusted OR (95% CI)	Age- and sex-adjusted POR (95% CI)
Age, mean (SE), years	56.95 (2.21)	1.10 (1.05, 1.14)	1.10 (1.05, 1.14)
Sex			
Male	13 (1.65)	1.00	1.00
Female	11 (1.33)	0.84 (0.37, 1.94)	0.78 (0.32, 1.75)
Canton†			
LUXEMBOURG	7 (1.65)	1.00	1.04 (0.60,2.05)
CLERVAUX	0 (0.00)	0.00 (0.00, Infinity)	0.87 (0.26, 1.79)
DIEKIRCH	1 (0.73)	0.70 (0.08, 5.87)	0.95 (0.46, 1.85)
REDANGE	1 (2.28)	1.57 (0.18, 13.60)	1.02 (0.48, 2.17)
VIANDEN	0 (0.00)	0.00 (0.00, Infinity)	0.90 (0.27, 2.31)
WILTZ	2 (4.24)	2.74 (0.52, 14.32)	1.15 (0.63, 3.07)
ECHTERNACH	0 (0.00)	0.00 (0.00, Infinity)	0.89 (0.27, 1.77)
GREVENMACHER	2 (2.46)	1.40 (0.28, 7.10)	1.08 (0.58, 2.69)
REMICH	3 (5.13)	4.19 (1.00, 17.65)	1.33 (0.72, 4.65)
CAPELLEN	2 (1.45)	0.77 (0.16, 3.83)	0.97 (0.49, 1.99)
ESCH/ALZETTE	5 (1.02)	0.65 (0.20, 2.10)	0.89 (0.43, 1.49)
MERSCH	1 (0.87)	0.54 (0.06, 4.56)	0.93 (0.43, 1.53)

† $p = 0.52$.

Table S16. Low glomerular filtration rate (chronic kidney disease) according to municipalities.

	Crude prevalence N (%)	Age- and sex-adjusted OR (95% CI)	Age- and sex-adjusted POR (95% CI)
Municipality †			
LUXEMBOURG	2 (0.85)	1.00	0.92 (0.42, 1.75)
BEAUFORT	0 (0.00)	0.00 (0.00, Infinity)	0.96 (0.31, 2.97)
BECH	0 (0.00)	0.00 (0.00, Infinity)	1.00 (0.35, 2.90)
BECKERICH	0 (0.00)	0.00 (0.00, Infinity)	0.98 (0.33, 2.80)
BERDORF	0 (0.00)	0.00 (0.00, Infinity)	0.96 (0.29, 2.64)
BERTRANGE	0 (0.00)	0.00 (0.00, Infinity)	0.92 (0.30, 2.11)
BETTEMBOURG	0 (0.00)	0.00 (0.00, Infinity)	0.86 (0.24, 2.00)
BETTENDORF	1 (6.93)	6.70 (0.51, 87.42)	1.08 (0.42, 3.32)
BETZDORF	0 (0.00)	0.00 (0.00, Infinity)	1.07 (0.41, 3.18)
BISSEN	0 (0.00)	0.00 (0.00, Infinity)	0.95 (0.30, 2.83)
BIWER	0 (0.00)	0.00 (0.00, Infinity)	1.01 (0.34, 2.82)
BOEVANGE/ATTERT	0 (0.00)	0.00 (0.00, Infinity)	0.97 (0.33, 2.50)
BOURSCHEID	0 (0.00)	0.00 (0.00, Infinity)	0.95 (0.34, 2.36)
BOUS	0 (0.00)	0.00 (0.00, Infinity)	1.26 (0.47, 5.52)
CLERVAUX	0 (0.00)	0.00 (0.00, Infinity)	0.92 (0.28, 2.45)
COLMAR-BERG	0 (0.00)	0.00 (0.00, Infinity)	0.93 (0.28, 2.28)
CONSDORF	0 (0.00)	0.00 (0.00, Infinity)	0.94 (0.30, 2.30)
CONTERN	1 (7.54)	6.08 (0.44, 83.88)	1.28 (0.58, 4.62)
DALHEIM	0 (0.00)	0.00 (0.00, Infinity)	1.22 (0.49, 4.25)
DIEKIRCH	0 (0.00)	0.00 (0.00, Infinity)	0.94 (0.29, 2.47)
DIFFERDANGE	1 (2.14)	2.86 (0.24, 34.22)	1.06 (0.38, 3.12)
DIPPACH	1 (5.77)	7.86 (0.60, 103.85)	1.08 (0.43, 3.15)
DUDELANGE	0 (0.00)	0.00 (0.00, Infinity)	0.76 (0.18, 1.56)
ECHTERNACH	0 (0.00)	0.00 (0.00, Infinity)	0.92 (0.29, 2.52)
ELL	0 (0.00)	0.00 (0.00, Infinity)	0.97 (0.25, 3.21)

ERPELDANGE	0 (0.00)	0.00 (0.00, Infinity)	0.91 (0.32, 2.35)
ESCH/ALZETTE	1 (1.12)	1.29 (0.11, 14.79)	0.88 (0.28, 1.99)
ESCH/SURE	0 (0.00)	0.00 (0.00, Infinity)	0.97 (0.33, 2.48)
ETTELBRUCK	0 (0.00)	0.00 (0.00, Infinity)	0.86 (0.23, 2.03)
FEULEN	0 (0.00)	0.00 (0.00, Infinity)	0.91 (0.24, 2.44)
FISCHBACH	0 (0.00)	0.00 (0.00, Infinity)	0.97 (0.36, 2.43)
FLAXWEILER	0 (0.00)	0.00 (0.00, Infinity)	1.13 (0.42, 3.97)
FRISANGE	1 (4.63)	10.60 (0.83, 135.82)	1.30 (0.49, 5.90)
GARNICH	0 (0.00)	0.00 (0.00, Infinity)	0.95 (0.31, 2.76)
GOESDORF	0 (0.00)	0.00 (0.00, Infinity)	0.99 (0.32, 2.82)
GREVENMACHER	0 (0.00)	0.00 (0.00, Infinity)	1.02 (0.39, 3.07)
GROSBOUS	0 (0.00)	0.00 (0.00, Infinity)	0.96 (0.32, 2.41)
HEFFINGEN	0 (0.00)	0.00 (0.00, Infinity)	0.99 (0.41, 2.66)
HESPERANGE	1 (2.73)	2.16 (0.18, 25.55)	1.10 (0.52, 3.25)
HOBSCHEID	0 (0.00)	0.00 (0.00, Infinity)	0.92 (0.28, 2.23)
JUNGLINSTER	2 (12.23)	17.13 (1.96, 149.51)	1.34 (0.65, 5.43)
KAERJEN	1 (3.44)	3.33 (0.28, 39.98)	1.06 (0.45, 2.83)
KAYL	0 (0.00)	0.00 (0.00, Infinity)	0.81 (0.21, 1.81)
KEHLEN	0 (0.00)	0.00 (0.00, Infinity)	0.91 (0.33, 2.00)
KIISCHPELT	0 (0.00)	0.00 (0.00, Infinity)	0.94 (0.30, 2.48)
KOERICH	0 (0.00)	0.00 (0.00, Infinity)	0.92 (0.28, 2.28)
KOPSTAL	0 (0.00)	0.00 (0.00, Infinity)	0.91 (0.31, 1.90)
LAC DE LA HAUTE SURE	1 (18.23)	23.44 (1.54, 357.98)	1.22 (0.50, 4.25)
LAROCHETTE	0 (0.00)	0.00 (0.00, Infinity)	0.93 (0.31, 2.35)
LENNINGEN	1 (12.87)	34.83 (2.44, 497.55)	1.40 (0.63, 6.62)
LEUDELANGE	0 (0.00)	0.00 (0.00, Infinity)	0.94 (0.29, 2.40)
LINTGEN	0 (0.00)	0.00 (0.00, Infinity)	0.92 (0.26, 2.63)
LORENTZWEILER	0 (0.00)	0.00 (0.00, Infinity)	0.96 (0.35, 2.39)
MAMER	0 (0.00)	0.00 (0.00, Infinity)	0.89 (0.32, 1.92)
MANTERNACH	0 (0.00)	0.00 (0.00, Infinity)	1.00 (0.32, 2.89)
MERSCH	1 (2.83)	2.69 (0.22, 32.49)	1.01 (0.42, 2.37)
MERTERT	0 (0.00)	0.00 (0.00, Infinity)	0.94 (0.25, 2.92)
MERTZIG	0 (0.00)	0.00 (0.00, Infinity)	0.95 (0.30, 2.46)
MOMPACH	0 (0.00)	0.00 (0.00, Infinity)	0.95 (0.28, 2.63)
MONDERCANGE	1 (4.26)	3.00 (0.25, 36.63)	1.01 (0.38, 2.94)
MONDORF-LES-BAINS	1 (8.07)	7.88 (0.57, 109.05)	1.43 (0.51, 7.98)
NIEDERANVEN	2 (7.49)	6.65 (0.82, 53.98)	1.31 (0.65, 4.39)
NOMMERN	0 (0.00)	0.00 (0.00, Infinity)	0.93 (0.30, 2.30)
PARC HOSINGEN	0 (0.00)	0.00 (0.00, Infinity)	0.89 (0.27, 2.38)
PETANGE	0 (0.00)	0.00 (0.00, Infinity)	0.88 (0.20, 2.60)
PREIZERDAUL	0 (0.00)	0.00 (0.00, Infinity)	0.98 (0.37, 2.85)
PUTSCHEID	0 (0.00)	0.00 (0.00, Infinity)	0.92 (0.27, 2.44)
RAMBROUCH	0 (0.00)	0.00 (0.00, Infinity)	0.94 (0.30, 2.38)
RECKANGE/MESS	0 (0.00)	0.00 (0.00, Infinity)	0.95 (0.29, 2.53)
REDANGE	0 (0.00)	0.00 (0.00, Infinity)	0.97 (0.31, 2.59)
REISDORF	0 (0.00)	0.00 (0.00, Infinity)	0.95 (0.29, 2.70)
REMICH	1 (12.98)	23.26 (1.58, 342.80)	1.50 (0.58, 8.96)
ROESER	0 (0.00)	0.00 (0.00, Infinity)	0.94 (0.29, 2.35)
ROSPORT	0 (0.00)	0.00 (0.00, Infinity)	0.96 (0.23, 3.26)
RUMELANGE	0 (0.00)	0.00 (0.00, Infinity)	0.83 (0.17, 2.12)
SAEUL	0 (0.00)	0.00 (0.00, Infinity)	0.98 (0.35, 2.62)
SANDWEILER	0 (0.00)	0.00 (0.00, Infinity)	1.05 (0.35, 2.93)
SANEM	1 (2.10)	2.11 (0.18, 24.72)	0.99 (0.40, 2.54)

SCHENGEN	0 (0.00)	0.00 (0.00, Infinity)	1.24 (0.48, 5.24)
SCHIEREN	0 (0.00)	0.00 (0.00, Infinity)	0.92 (0.26, 2.33)
SCHIFFLANGE	0 (0.00)	0.00 (0.00, Infinity)	0.83 (0.22, 2.09)
SCHUTTRANGE	1 (8.12)	26.19 (1.83, 375.71)	1.33 (0.58, 5.47)
STADTBREDIMUS	0 (0.00)	0.00 (0.00, Infinity)	1.27 (0.47, 5.78)
STEINFORT	0 (0.00)	0.00 (0.00, Infinity)	0.93 (0.29, 2.51)
STEINSEL	0 (0.00)	0.00 (0.00, Infinity)	0.95 (0.34, 2.41)
STRASSEN	0 (0.00)	0.00 (0.00, Infinity)	0.92 (0.33, 2.21)
TANDEL	0 (0.00)	0.00 (0.00, Infinity)	0.92 (0.26, 2.72)
TROISVIERGES	0 (0.00)	0.00 (0.00, Infinity)	0.91 (0.18, 3.27)
TUNTANGE	0 (0.00)	0.00 (0.00, Infinity)	0.96 (0.31, 2.54)
USELDANGE	1 (21.13)	39.56 (2.51, 624.77)	1.14 (0.48, 4.00)
VALLéE DE L'ERNZ	0 (0.00)	0.00 (0.00, Infinity)	0.93 (0.33, 2.29)
VIANDEN	0 (0.00)	0.00 (0.00, Infinity)	0.92 (0.21, 3.17)
WAHL	0 (0.00)	0.00 (0.00, Infinity)	0.96 (0.25, 3.13)
WALDBILLIG	0 (0.00)	0.00 (0.00, Infinity)	0.95 (0.34, 2.45)
WALDBREDIMUS	0 (0.00)	0.00 (0.00, Infinity)	1.22 (0.49, 4.68)
WALFERDANGE	0 (0.00)	0.00 (0.00, Infinity)	0.88 (0.28, 2.13)
WEILER-LA-TOUR	0 (0.00)	0.00 (0.00, Infinity)	1.12 (0.43, 3.33)
WEISWAMPACH	0 (0.00)	0.00 (0.00, Infinity)	0.94 (0.23, 2.97)
WILTZ	0 (0.00)	0.00 (0.00, Infinity)	0.95 (0.30, 2.40)
WINCRANGE	0 (0.00)	0.00 (0.00, Infinity)	0.97 (0.27, 3.19)
WINSELER	1 (17.71)	35.53 (1.72, 733.57)	1.20 (0.47, 5.29)
WORMELDANGE	0 (0.00)	0.00 (0.00, Infinity)	1.18 (0.42, 4.74)

† $p = 1.00$.

Table S17. Low physical activity according to cantons.

	**Crude prevalence N (%)**	**Age- and sex-adjusted OR (95% CI)**	**Age- and sex-adjusted POR (95% CI)**
Age, mean (SE), years	42.32 (0.15)	1.01(0.99,1.02)	1.00(0.99,1.02)
Sex			
Male	592 (88.70)	1.00	1.00
Female	633 (90.27)	1.06 (0.75, 1.52)	1.09 (0.75, 1.54)
Canton†			
LUXEMBOURG	345 (93.41)	1.00	1.47 (0.98, 2.34)
CLERVAUX	34 (81.67)	0.40 (0.15, 1.04)	0.83 (0.45, 1.37)
DIEKIRCH	74 (84.88)	0.58 (0.26, 1.29)	0.96 (0.63, 1.48)
REDANGE	34 (88.37)	0.58 (0.19, 1.78)	1.01 (0.62, 1.73)
VIANDEN	7 (70.53)	0.12 (0.03, 0.44)	0.64 (0.23, 1.20)
WILTZ	38 (86.21)	0.44 (0.17, 1.15)	0.89 (0.50, 1.55)
ECHTERNACH	36 (83.70)	0.36 (0.15, 0.90)	0.87 (0.48, 1.37)
GREVENMACHER	67 (92.47)	0.92 (0.34, 2.51)	1.22 (0.77, 2.25)
REMICH	47 (93.05)	0.83 0.27, 2.49)	1.25 (0.75, 2.52)
CAPELLEN	118 (93.58)	0.90 (0.41, 2.00)	1.29 (0.85, 2.17)
ESCH/ALZETTE	355 (88.07)	0.51 (0.31, 0.86)	0.96 (0.67, 1.32)
MERSCH	70 (84.20)	0.37 (0.18, 0.77)	0.88 (0.56, 1.34)

† $p = 0.03$.

Table S18. Low physical activity according to municipalities.

	Crude prevalence N (%)	Age- and sex-adjusted OR (95% CI)	Age- and sex-adjusted POR (95% CI)
Municipality †			
LUXEMBOURG	185 (96.03)	1.00	1.85 (1.06, 3.45)
BEAUFORT	2 (50.32)	0.04 (0.01, 0.35)	0.69 (0.23, 1.61)
BECH	0 (0.00)	0.00 (0.00, Infinity)	0.87 (0.32, 1.93)
BECKERICH	8 (100.00)	4234586.32 (0.00, Infinity)	1.17 (0.53, 3.13)
BERDORF	4 (61.00)	0.08 (0.01, 0.50)	0.75 (0.25, 1.67)
BERTRANGE	23 (85.49)	0.22 (0.06, 0.80)	0.98 (0.45, 1.99)
BETTEMBOURG	24 (93.20)	0.44 (0.09, 2.24)	1.11 (0.56, 2.55)
BETTENDORF	9 (84.94)	0.32 (0.04, 2.89)	0.90 (0.37, 2.25)
BETZDORF	6 (100.00)	3997918.69 (0.00, Infinity)	1.20 (0.54, 2.88)
BISSEN	5 (100.00)	4657327.57 (0.00, Infinity)	1.07 (0.48, 2.66)
BIWER	6 (100.00)	4213680.86 (0.00, Infinity)	1.18 (0.53, 3.14)
BOEVANGE/ATTERT	3 (65.66)	0.11 (0.01, 1.24)	0.88 (0.36, 2.14)
BOURSCHEID	0 (0.00)	0.00 (0.00, Infinity)	0.69 (0.24, 1.56)
BOUS	2 (100.00)	4389738.91 (0.00, Infinity)	1.17 (0.45, 3.05)
CLERVAUX	13 (87.83)	0.49 (0.06, 4.31)	0.93 (0.40, 2.01)
COLMAR-BERG	10 (100.00)	4565984.81 (0.00, Infinity)	1.15 (0.55, 2.68)
CONSDORF	4 (84.36)	0.14 (0.01, 1.45)	0.89 (0.39, 2.09)
CONTERN	11 (86.78)	0.41 (0.05, 3.66)	1.16 (0.52, 2.69)
DALHEIM	8 (100.00)	4459243.79 (0.00, Infinity)	1.25 (0.57, 2.97)
DIEKIRCH	15 (81.45)	0.29 (0.05, 1.51)	0.89 (0.43, 2.02)
DIFFERDANGE	36 (93.16)	0.46 (0.11, 1.89)	1.23 (0.65, 2.70)
DIPPACH	15 (100.00)	4451052.41 (0.00, Infinity)	1.38 (0.67, 3.56)
DUDELANGE	53 (85.23)	0.24 (0.08, 0.70)	0.87 (0.46, 1.60)
ECHTERNACH	13 (100.00)	4412473.99 (0.00, Infinity)	1.21 (0.52, 3.35)
ELL	1 (100.00)	4618407.71 (0.00, Infinity)	0.98 (0.40, 2.74)
ERPELDANGE	4 (100.00)	4625409.16 (0.00, Infinity)	1.01 (0.45, 2.36)
ESCH/ALZETTE	66 (86.32)	0.25 (0.09, 0.69)	0.88 (0.48, 1.51)
ESCH/SURE	7 (100.00)	4564347.81 (0.00, Infinity)	1.01 (0.48, 2.68)
ETTELBRUCK	24 (96.93)	0.90 (0.11, 7.61)	1.17 (0.60, 2.74)
FEULEN	2 (100.00)	3866904.34 (0.00, Infinity)	0.98 (0.39, 2.59)
FISCHBACH	3 (100.00)	4289417.06 (0.00, Infinity)	1.01 (0.44, 2.30)
FLAXWEILER	4 (100.00)	4100318.44 (0.00, Infinity)	1.23 (0.51, 3.35)
FRISANGE	14 (78.80)	0.18 (0.04, 0.77)	0.92 (0.41, 2.06)
GARNICH	4 (100.00)	4169862.26 (0.00, Infinity)	1.26 (0.54, 3.57)
GOESDORF	7 (85.30)	0.26 (0.03, 2.45)	0.86 (0.37, 1.99)
GREVENMACHER	18 (96.69)	0.69 (0.08, 5.90)	1.26 (0.61, 3.18)
GROSBOUS	3 (100.00)	4308231.63 (0.00, Infinity)	0.99 (0.40, 2.60)
HEFFINGEN	1 (100.00)	4864832.56 (0.00, Infinity)	0.98 (0.40, 2.44)
HESPERANGE	36 (97.85)	1.34 (0.16, 11.22)	1.49 (0.78, 3.56)
HOBSCHEID	10 (100.00)	4306099.55 (0.00, Infinity)	1.26 (0.59, 3.40)
JUNGLINSTER	13 (77.25)	0.16 (0.04, 0.71)	0.88 (0.39, 1.67)
KAERJEN	25 (89.74)	0.31 (0.07, 1.26)	1.10 (0.51, 2.39)
KAYL	27 (85.83)	0.26 (0.07, 0.94)	0.92 (0.44, 1.97)
KEHLEN	12 (86.26)	0.22 (0.04, 1.17)	0.95 (0.42, 2.07)
KIISCHPELT	1 (100.00)	3917443.95 (0.00, Infinity)	0.88 (0.35, 2.29)
KOERICH	12 (93.41)	0.45 (0.05, 3.94)	1.16 (0.54, 2.66)
KOPSTAL	9 (81.45)	0.15 (0.03, 0.85)	0.93 (0.40, 2.13)
LAC DE LA HAUTE SURE	5 (76.49)	0.18 (0.02, 1.80)	0.85 (0.34, 2.04)
LAROCHETTE	9 (81.48)	0.17 (0.03, 0.96)	0.85 (0.37, 1.86)

LENNINGEN	9 (100.00)	4564446.88 (0.00, Infinity)	1.34 (0.61, 3.63)
LEUDELANGE	3 (100.00)	4557330.42 (0.00, Infinity)	1.15 (0.43, 2.76)
LINTGEN	8 (88.66)	0.28 (0.03, 2.57)	0.95 (0.38, 2.30)
LORENTZWEILER	6 (78.15)	0.10 (0.02, 0.60)	0.83 (0.35, 1.72)
MAMER	21 (96.51)	0.78 (0.09, 6.69)	1.30 (0.63, 2.92)
MANTERNACH	3 (100.00)	4855491.46 (0.00, Infinity)	1.15 (0.48, 2.95)
MERSCH	21 (78.61)	0.13 (0.04, 0.42)	0.72 (0.33, 1.35)
MERTERT	12 (90.28)	0.44 (0.05, 3.86)	1.16 (0.49, 3.11)
MERTZIG	3 (65.50)	0.12 (0.01, 1.31)	0.91 (0.37, 2.06)
MOMPACH	2 (61.50)	0.07 (0.01, 0.88)	0.93 (0.33, 2.63)
MONDERCANGE	17 (95.18)	0.60 (0.07, 5.17)	1.16 (0.55, 2.94)
MONDORF-LES-BAINS	11 (100.00)	4337942.11 (0.00, Infinity)	1.37 (0.58, 3.94)
NIEDERANVEN	20 (92.93)	0.37 (0.07, 1.91)	1.11 (0.55, 2.24)
NOMMERN	3 (100.00)	4570804.18 (0.00, Infinity)	1.00 (0.42, 2.38)
PARC HOSINGEN	10 (100.00)	4437637.29 (0.00, Infinity)	1.07 (0.46, 2.83)
PETANGE	33 (92.21)	0.43 (0.11, 1.76)	1.22 (0.59, 3.08)
PREIZERDAUL	1 (59.60)	0.04 (0.00, 0.64)	0.82 (0.32, 1.97)
PUTSCHEID	2 (100.00)	4196640.26 (0.00, Infinity)	0.89 (0.35, 2.43)
RAMBROUCH	5 (83.19)	0.18 (0.02, 1.72)	0.89 (0.37, 2.02)
RECKANGE/MESS	10 (100.00)	4500444.45 (0.00, Infinity)	1.31 (0.59, 3.19)
REDANGE	7 (78.71)	0.13 (0.02, 0.75)	0.82 (0.35, 1.84)
REISDORF	3 (100.00)	4763503.42 (0.00, Infinity)	0.92 (0.35, 2.30)
REMICH	6 (100.00)	4203834.36 (0.00, Infinity)	1.27 (0.48, 3.85)
ROESER	15 (78.16)	0.11 (0.03, 0.39)	0.77 (0.32, 1.49)
ROSPORT	9 (100.00)	4512666.33 (0.00, Infinity)	1.21 (0.51, 3.45)
RUMELANGE	7 (83.45)	0.26 (0.03, 2.45)	0.95 (0.40, 2.64)
SAEUL	1 (100.00)	4617983.58 (0.00, Infinity)	1.03 (0.42, 2.78)
SANDWEILER	8 (80.12)	0.15 (0.03, 0.86)	0.97 (0.40, 2.30)
SANEM	38 (91.56)	0.35 (0.10, 1.27)	1.10 (0.60, 2.13)
SCHENGEN	9 (79.44)	0.12 (0.03, 0.53)	0.88 (0.34, 2.29)
SCHIEREN	4 (100.00)	4242153.99 (0.00, Infinity)	1.07 (0.50, 2.73)
SCHIFFLANGE	12 (78.12)	0.11 (0.03, 0.43)	0.71 (0.29, 1.52)
SCHUTTRANGE	10 (87.72)	0.39 (0.04, 3.51)	1.17 (0.52, 2.83)
STADTBREDIMUS	1 (100.00)	5444797.76 (0.00, Infinity)	1.19 (0.44, 3.17)
STEINFORT	10 (100.00)	4724973.95 (0.00, Infinity)	1.32 (0.60, 3.49)
STEINSEL	10 (77.73)	0.12 (0.03, 0.53)	0.83 (0.33, 1.63)
STRASSEN	11 (100.00)	4361479.55 (0.00, Infinity)	1.30 (0.61, 3.28)
TANDEL	3 (49.85)	0.03 (0.01, 0.15)	0.55 (0.18, 1.27)
TROISVIERGES	8 (80.55)	0.15 (0.03, 0.86)	0.68 (0.23, 1.63)
TUNTANGE	1 (31.98)	0.04 (0.00, 0.71)	0.88 (0.31, 2.01)
USELDANGE	5 (100.00)	4278548.69 (0.00, Infinity)	1.07 (0.47, 2.65)
VALLéE DE L'ERNZ	10 (76.24)	0.20 (0.04, 1.10)	0.86 (0.38, 1.80)
VIANDEN	2 (100.00)	3977188.77 (0.00, Infinity)	0.88 (0.26, 2.37)
WAHL	3 (100.00)	4260378.22 (0.00, Infinity)	0.98 (0.39, 2.54)
WALDBILLIG	2 (100.00)	4255189.56 (0.00, Infinity)	0.97 (0.39, 2.56)
WALDBREDIMUS	1 (43.02)	0.03 (0.00, 0.61)	0.99 (0.35, 2.50)
WALFERDANGE	27 (91.29)	0.34 (0.08, 1.39)	1.08 (0.56, 2.40)
WEILER-LA-TOUR	4 (100.00)	4384934.19 (0.00, Infinity)	1.19 (0.52, 3.00)
WEISWAMPACH	0 (0.00)	0.00 (0.00, Infinity)	0.67 (0.19, 1.92)
WILTZ	14 (81.47)	0.13 (0.03, 0.51)	0.68 (0.30, 1.41)
WINCRANGE	3 (51.41)	0.06 (0.01, 0.41)	0.64 (0.22, 1.42)
WINSELER	4 (100.00)	4324049.12 (0.00, Infinity)	0.89 (0.36, 2.36)
WORMELDANGE	5 (100.00)	4298265.97 (0.00, Infinity)	1.24 (0.50, 3.27)

† $p < 0.001$.

BAYESIAN GEO-ADDITIVE REGRESSION MODELS

Spatial analyses of cardiovascular disease (CVD) often are confined to using region-specific dummy variables to capture the spatial dimension. Here, we go a step further by exploring regional patterns of CVD and, possibly nonlinear, effects of other factors within a simultaneous, coherent regression framework using a geo-additive semi-parametric mixed model. Because the predictor contains usual linear terms, nonlinear effects of metrical covariates and geographic effects in additive form, such models are also called geo-additive models. Kammann and Wand [1] proposed this type of model within an empirical Bayesian approach. Here, we apply a fully Bayesian approach as suggested in Fahrmeir and Lang [2] which is based on Markov priors and uses MCMC techniques for inference and model checking.

Classical linear regression models of the form

$$y_i = w_i'\gamma + \varepsilon_i, \qquad \varepsilon_i \sim N(0,\sigma^2), \tag{1}$$

(y_i, w_i), i = 1,....,n, on a response variable y and a vector w of covariates assume that the mean $E\,(y_i \mid w_i)$ can be modeled through a *linear predictor* $w_i'\gamma$. In our application to

FGM and in many other regression situations, we are facing the following problems: First, for the *continuous covariates* in the data set, the assumption of a strictly linear effect on the response y may not be appropriate. In our study, such covariate is the respondent's age. Generally, it will be difficult to model the possibly nonlinear effect of such covariates through a parametric functional form, which has to be *linear* in the parameters, prior to any data analysis.

Second, in addition to usual covariates, geographical small-area information was given in form of a location variable s, indicating the region, department or community where individuals or units in the sample size live or come from. In our study, this geographical information is given by the regions in Senegal. Attempts to include such small-area information using region/commune-specific dummy-variables would in our case entail more than 105 dummy-variables for the communes and 10 dummies for the regions and using this approach we would not assess spatial inter-dependence. The latter problem cannot also be resolved through conventional multilevel modeling using uncorrelated random effects [3]. It is reasonable to assume that areas close to each other are more similar than areas far apart, so that spatially correlated random effects are required.

To overcome these difficulties, we replace the strictly linear predictor $\eta_i = x_i'\beta + w_i'\gamma + \varepsilon_i$

With a logit link function with dynamic and spatial effects, Pr(y_i=1 | η_i)=e^{η_i} /(1+ e^{η_i}), and a geoadditive semi-parametric predictor μ_i = h(η_i):

$$\eta_i = f_1(x_{i1}) + \ldots + f_p(x_{ip}) + f_{spat}(s_i) + w'_i\,\gamma + \varepsilon_i \tag{2}$$

where h is a known response function with a logit link function, f_1, …, f_p are non-linear smoothed effects of the metrical covariates (respondent's age), and $f_{spat}(s_i)$ is the effect of the spatial covariate $s_i \in \{1,...,S\}$ labelling the region in Senegal. Covariates in w'_i are usual categorical variables such as gender and urban-rural residence. Regression models with predictors as in (2) are sometimes referred to as geo-additive models. The observation model (2) may be extended by including interaction $f(x)w$ between a continuous covariate x and a binary component of w, say, leading to so called varying coefficient models, or by adding a nonlinear interaction $f_{1,2}(x_1, x_2)$ of two continuous covariates.

In a further step, we may split up the spatial effect fspat into a spatially correlated (structured) and an uncorrelated (unstructured) effect

$$f_{spat}(s_i) = f_{str}(s_i)\, f_{untsr}(s_i)$$

A rationale is that a spatial effect is usually a surrogate of many unobserved influences, some of them may obey a strong spatial structure and others may be present only locally. By estimating a structured and an unstructured effect, we aim at separating between the two kinds of factors. As a side effect, we are able to assess to some extent the amount of spatial dependency in the data by

observing which one of the two effects is larger. If the unstructured effect exceeds the structured effect, the spatial dependency is smaller and vice versa. It should be noted that all functions are centred about zero for identification purpose, thus fixed effects parameters automatically include an intercept term γ_0.

In a Bayesian approach unknown functions f_j and parameters γ as well as the variance parameter σ^2 are considered as random variables and have to be supplemented with appropriate prior assumptions. In the absence of any prior knowledge we assume independent diffuse priors $\gamma_j \propto const$, $j=1,\ldots,r$ for the parameters of fixed effects. Another common choice is highly dispersed Gaussian priors.

Several alternatives are available as smoothness priors for the unknown functions $f_j\,(x_j)$, see Fahrmeir and Lang [2], Belitz, Brezger and Kneib [4]. We use Bayesian P(enalized)-Splines, introduced by Eilers and Marx [5] in a frequentist setting. It is assumed that an unknown smooth function $f_j\,(x_j)$ can be approximated by a polynomial spline of low degree. The usual choices are cubic splines, which are twice continuously differentiable piecewise cubic polynomials defined for a grid of k equally spaced knot p on the relevant interval $[a,b]$ of the x-axis. Such a spline can be written in terms of a linear combination B-spline basis functions $B_m(x)$, i.e.,

$$f(x)=\sum_{m=1}^{l}\beta_m B_m(x) \tag{3}$$

These basis functions have finite support on four neighbouring intervals of the grid, and are zero elsewhere. A comparably small number of knots (usually between 10 and 40) is chosen to ensure enough flexibility in combination with a roughness penalty based on second order difference of adjacent B-spline coefficients to guarantee sufficient smoothness of the fitted curves. In our

Bayesian approach this corresponds to second order random walks

$$\beta_m = 2\beta_{m-1} - \beta_{m-2} + u_m \tag{4}$$

with Gaussian errors $u_m \sim N(0,\tau^2)$. The variance parameter τ^2 controls the amount of smoothness, and is also estimated from the data. More details on Bayesian P-Splines can be found in Lang and Brezger [6]. Note that random walks are the special case of B-Splines of degree zero.

We now turn our attention to the spatial effects f_{str} and f_{unstr}. For the spatially correlated effect f_{str} (s), s = 1, … S, we choose Markov random field priors common in spatial statistics [7]. These priors reflect spatial neighbourhood relationships. For geographical data one usually assumes that two sites or regions s and r are neighbours if they share a common boundary. Then a spatial extension of random walk models leads to the conditional, spatially autoregressive specification

$$f_{str}(s)\mid f_{str}(r), r \neq s \sim N\Big(\sum_{r\in\partial_s} f_{str}(r)/N_s, \tau^2/N_s\Big) \tag{5}$$

where N_s is the number of adjacent regions, and $r \in \partial_s$ denotes that region r is a neighbour of region s. Thus the (conditional) mean of $f_{str}(s)$ is an average of function evaluations $f_{str}(s)$ of neighbouring regions. Again the variance τ^2_{str} controls the degree of smoothness.

For a spatially uncorrelated (unstructured) effect f_{unstr} a common assumption is that the parameters $f_{unstr}(s)$ are i.i.d. Gaussian

$$f_{unstr}(s) \mid \tau^2_{unstr} \sim N(0, \tau^2_{unstr}) \tag{6}$$

Variance or smoothness parameters τ^2_j, $j=1,\ldots,p$, str, $unstr$, are also considered as unknown and estimated simultaneously with corresponding unknown functions f_j. Therefore, hyperpriors are assigned to them in a second stage of the hierarchy by highly dispersed inverse gamma distributions $p(\tau^2_j) \sim IG(a_j,b_j)$ with known hyper-parameters a_j and b_j. Standard choices for the hyperparameters are a =1 and b =0.005 or a = b = 0,001. Jeffrey's noninformative prior is closer to the later choice, and since practical experience shows that regression parameters depend on the choice of

hyperparameters, we have investigated in our application the sensitivity to this choice. Since some regions in Senegal do not have many neighbours, we have investigated the sensibility of the choice of Markov Random Field (MRF) prior with other priors supported by BayesX such as Gaussian random field (GRF) priors, but the resulting maps from the two priors did not differ much. Therefore, we considered the MRF prior for the spatial effects. For model choice, we routinely used the Deviance Information Criterion (DIC) developed in Spiegelhalter et. al. [8] as a measure of fit and model complexity. Before commenting on the substantive results, it is important to point out this model had the best fit after evaluation of the fit criteria using Deviance Information Criteria (DIC).

The model assumes that $f_1(\ )$, $f_2(\ )$ *and* f_{str} are nonlinear effects and spatial effects were the same in all the country. This was confirmed by prior separate analyses of the non-linear effects in other countries, which were found to be remarkably similar. The analysis was carried out using BayesX version 0.9, software for Bayesian inference based on Markov Chain Monte Carlo simulation techniques.

Quite clearly, the methods used here are able to identify more subtle socioeconomic and spatial influences on CVD than reliance on linear models with regional dummy variables. As such, they are useful for diagnostic purposes to identify the need to find additional variables that can account for this spatial structure. Moreover, even if the causes of spatial structures are not fully explained, one can use this spatial information for campaigns to eliminate the practice of FGM and planning purposes, which is gaining increasing importance in policy circles, that attempt to focus the allocation of public resources to the most at high risk population.

Multivariate Bayesian geo-additive regression models were used to evaluate the significance of the POR determined for the fixed effects and spatial effects between prevalence of CVD in Luxembourg. Each factor was looked at separately in unadjusted models using conventional logistic regression models. Next, fully adjusted multivariate Bayesian geo-additive regressions analyses were performed to look again for a statistically significant correlation between these variables, but this time further controlling for any influence from individual (age), ethnicity, education and religious factors. A P-value of <0.05 was considered indicative of a statistically significant difference.

References

1. Kammann E.E.; Wand M.P. Geoadditive Models. *J. R. Stat. Soc. C* **2003**, *52*, 1–18.
2. Fahrmeir, L.; Lang, S. Bayesian inference for generalized additive mixed models based on Markov random field priors. *Appl. Stat.* **2001**, *50*, 201–220.
3. Kandala N.B.; Tigbe, W.; Manda, S.O.; Stranges, S. Geographic variation of hypertension in sub-saharan Africa: a case study of South Africa. *Am. J. Hypertens*. **2013**, *26*, 382–391.
4. Belitz, C.; Brezger, A.; Kneib, T.; Lang, S. BayesX Software for Bayesian Inference in Structured Additive Regression Models Version 2.0.1. **2012**. Available online: http://www. stat. uni-muenchen. de/~ bayesx/manual/methodology_manual. pdf (accessed on 5 June 2017).
5. Eilers, P.H.; Marx, B.D. Flexible smoothing with *B*-splines and penalties. *Stat. Sci.* **1996**, *11*, 89–121.
6. Lang, S., Brezger, A. Bayesian P-Splines. *J. Comput. Graph. Stat.* **2004**, 13, 183–212.
7. Besag, J.E.; York, J.C.; Molli´e, A. Bayesian image restoration, with two applications in spatial statistics (with Discussion). *Ann. Inst. Stat. Math.* **1991**, *43*, 1–59**.**
8. Spiegelhalter, D.; Best, N.; Carlin, B.; Van der Line, A. Bayesian measures of models complexity and fit. *J. R. Stat. Soc.* **2002**, *B*, 1–34.
